# The Zic family homologue Odd-paired regulates *Alk* expression in *Drosophila*

**DOI:** 10.1371/journal.pgen.1006617

**Published:** 2017-04-03

**Authors:** Patricia Mendoza-García, Fredrik Hugosson, Mahsa Fallah, Michael L. Higgins, Yasuno Iwasaki, Kathrin Pfeifer, Georg Wolfstetter, Gaurav Varshney, Dmitry Popichenko, J. Peter Gergen, Korneel Hens, Bart Deplancke, Ruth H. Palmer

**Affiliations:** 1 Department of Medical Biochemistry and Cell Biology, Institute of Biomedicine, Sahlgrenska Academy, University of Gothenburg, Gothenburg, Sweden; 2 Department of Molecular Biology, Umeå University, Umeå, Sweden; 3 Department of Biochemistry and Cell Biology, Stony Brook University, Stony Brook, New York, United States of America; 4 Centre for Neural Circuits and Behaviour, University of Oxford, Oxford, United Kingdom; 5 Laboratory of Systems Biology and Genetics, Lausanne, Switzerland; University of Michigan Medical School, UNITED STATES

## Abstract

The Anaplastic Lymphoma Kinase (Alk) receptor tyrosine kinase (RTK) plays a critical role in the specification of founder cells (FCs) in the *Drosophila* visceral mesoderm (VM) during embryogenesis. Reporter gene and CRISPR/Cas9 deletion analysis reveals enhancer regions in and upstream of the *Alk* locus that influence tissue-specific expression in the amnioserosa (AS), the VM and the epidermis. By performing high throughput yeast one-hybrid screens (Y1H) with a library of *Drosophila* transcription factors (TFs) we identify Odd-paired (Opa), the *Drosophila* homologue of the vertebrate Zic family of TFs, as a novel regulator of embryonic *Alk* expression. Further characterization identifies evolutionarily conserved Opa-binding *cis*-regulatory motifs in one of the *Alk* associated enhancer elements. Employing *Alk* reporter lines as well as CRISPR/Cas9-mediated removal of regulatory elements in the *Alk* locus, we show modulation of *Alk* expression by Opa in the embryonic AS, epidermis and VM. In addition, we identify enhancer elements that integrate input from additional TFs, such as Binou (Bin) and Bagpipe (Bap), to regulate VM expression of *Alk* in a combinatorial manner. Taken together, our data show that the Opa zinc finger TF is a novel regulator of embryonic *Alk* expression.

## Author summary

The Alk receptor tyrosine kinase is employed repeatedly during *Drosophila* development to drive signaling events in a variety of tissues. The spatial and temporal expression pattern of the *Alk* gene is tightly regulated. Identifying factors that influence the expression of *Alk* is important to better understand how Alk signaling is controlled. In this paper we characterize *cis*-regulatory sequences in the *Alk* locus and the transcription factors that bind them to govern *Alk* expression in the *Drosophila* embryo. Using a robotic protein-DNA interaction assay, we identified the Zic family transcription factor Odd-paired as a factor that binds to regulatory elements in the *Alk* locus. Binding of Odd-paired to *Alk* *cis*-regulatory elements varies spatially, revealing a requirement for additional transcription factors such as the NK3 and FoxF orthologues Bagpipe and Biniou in a subset of *Alk*-expressing tissues. Our findings provide new insight into the dynamics underlying temporal and spatial regulation of the *Alk* receptor during embryogenesis.

## Introduction

During embryogenesis, the Anaplastic Lymphoma Kinase (Alk) receptor tyrosine kinase (RTK) is dynamically expressed predominantly in the primordia of the visceral mesoderm (VM), the developing CNS, the amnioserosa (AS) and in a restricted manner in the epidermis [1]. Alk plays a critical role during VM development, where it is activated in response to the secreted ligand Jelly Belly (Jeb) driving the Ras/MAPK/ERK pathway [2–5]. This leads to expression of founder cell (FC) specific transcription factors (TFs) such as *Hand* [6], *optomotor-blind related-1* (*org-1*) [4] and factors important in the muscle cell fusion process like *dumbfounded/kin of irre* (*duf/kirre*) [3–5]. Jeb/Alk signaling also leads to downregulation of fusion competent myoblast (FCM)-specific factors such as *sticks and stones* (*sns*) [7] and *Verprolin 1* (*vrp1*) [8–10]. In addition, Alk signaling in the VM modulates the subcellular localization of the Gli-family TF Lame duck (Lmd), resulting in Lmd translocation from the nucleus to the cytoplasm [11]. Thus, signaling regulated by Jeb/Alk is critical for embryonic FC-specification and the subsequent fusion with FCMs to form a functional larval midgut muscle [2–5].

While we and others have previously identified and characterized several important components and targets of the Alk RTK signaling pathway, little is currently understood about the molecular mechanisms regulating the spatial and temporal expression of the Alk receptor itself. Development of the early VM requires the activity of the NK4/msh-2-like homeobox TF Tinman (Tin) for dorsal mesoderm differentiation, as well as the NK3 and FoxF orthologues Bagpipe (Bap) and Biniou (Bin) [12–15]. Interestingly, the expression patterns of *bap* and *bin* in the VM primordia are similar to that of *Alk* [15]. In addition, ChIP-on-chip studies have shown the region upstream of *Alk* gene to be occupied by several mesodermally expressed TFs, such as Bin, Bap, Twist (Twi), Tin and Myocyte enhancer factor 2 (Mef2) at different time points during embryogenesis [16, 17]. While binding of these factors has been documented, their importance in the regulation of *Alk* transcription in the VM has only been initially characterized in case of Tin [16, 17].

Here we address regulation of *Alk* expression during embryogenesis. We have employed a combination of *in vitro* and *in vivo* approaches to identify and characterize *Alk*-specific enhancer elements, including high throughput yeast one-hybrid screening (Y1H) with a library of *Drosophila* TFs [18]. This Y1H screen identified the zinc finger TF Odd-paired (Opa) as binding to an evolutionary conserved *cis*-regulatory module (CRM) within one of the *Alk*-associated enhancer regions. In agreement with these findings, *opa* mutants displayed a complete loss of *Alk* expression in the epidermis and reduced levels of Alk in the VM. Furthermore, CRISPR/Cas9-mediated deletion of the Opa binding site containing region in the *Alk* locus resulted in a reduction of VM Alk protein together with loss of *Alk* expression in both the AS and embryonic epidermis, indicating that Opa plays an important role in tissue-specific *Alk* expression during embryogenesis. We have also identified additional enhancer regions regulated by the Bin and Bap TFs, likely together with additional TFs, that work with the Opa binding CRM to regulate *Alk* expression in the VM in a combinatorial manner.

## Results

### Identification of regulatory regions involved in *Alk* expression during embryogenesis

**Fig 1:**
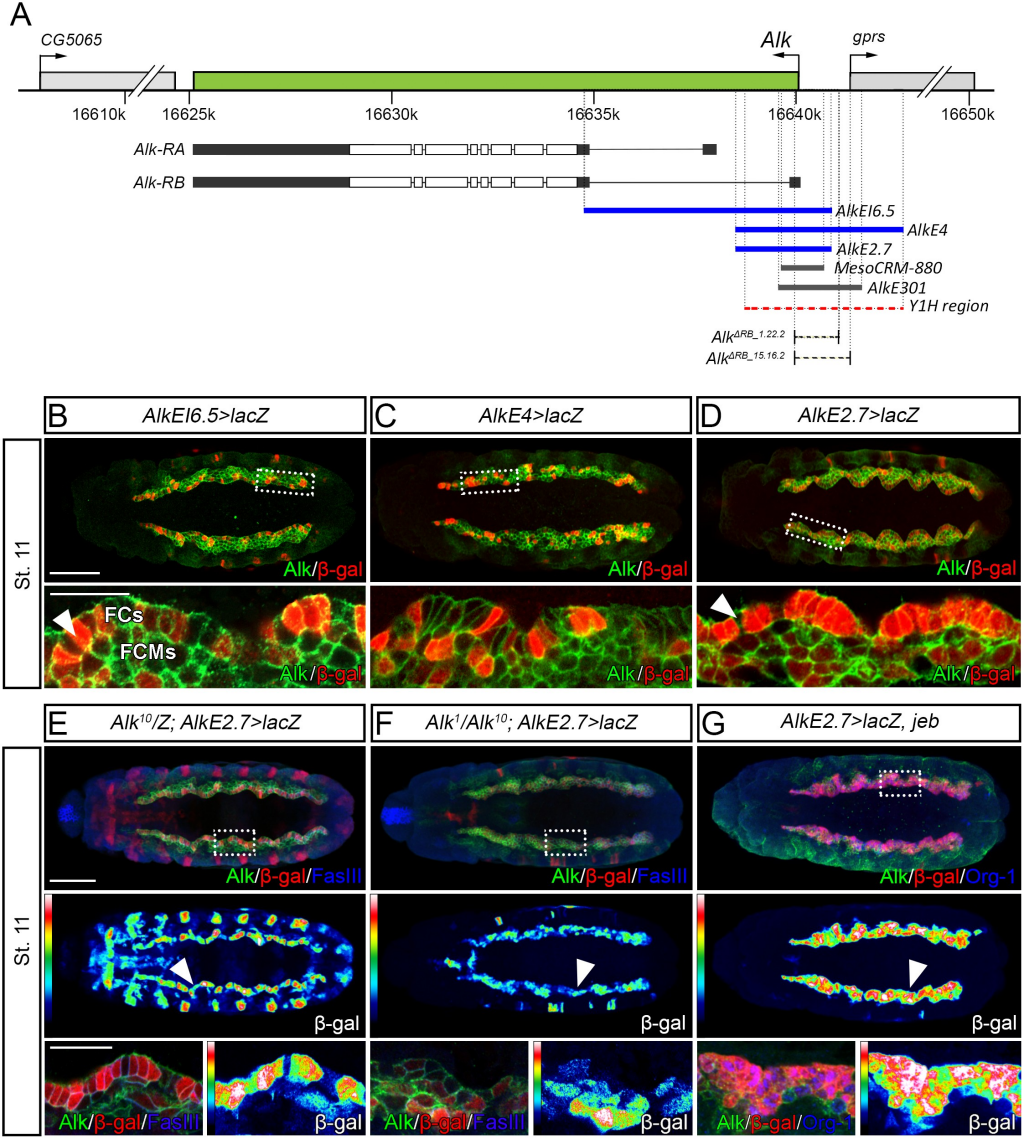
Reporter gene analysis identifies putative regulatory elements responsible for *Alk* expression. **(A)** Genomic organization of the *Alk* locus (*green*) and the neighboring genes *CG5065* and *gprs* (*light gray*). Intron-exon structure of both *Alk-RA* and *Alk-RB* transcripts (*Alk* open reading frame in *white*) and the analyzed reporter constructs (*blue lines*) are shown below. The *MesoCRM-880* and *AlkE301* CRMs identified in previous ChIP analyses are depicted as *grey* lines and the 3.6 kb region subjected to Y1H analysis is shown in *red* (*dashed lines*). CRISPR/Cas9 deletions disrupting the *Alk-RB* promoter, *Alk*^*ΔRB_1*.*22*.*2*^ and *Alk*^*ΔRB_15*.*16*.*2*^, are indicated as *black dashed lines*. **(B)** *AlkEI6*.*5* drives expression in the trunk VM at stage 11; with strongest expression in the founder cells (FCs) (close up; FCs, *arrowhead*). **(C)** *AlkE4* shows a slightly more restricted VM expression pattern compared to that of *AlkEI6*.*5*. **(D)** Similarly, *AlkE2*.*7* is expressed in the entire VM with marked stronger expression in the FCs (close up, *arrowhead*). **(E-F)** *AlkE2*.*7* expression in the FCs is responsive to Alk signaling. *lacZ* expression in *Alk*^*1*^*/Alk*^*10*^ embryos is weaker when compared to *Alk*^*10*^ heterozygote balanced controls (*arrowhead*; compare β-gal *heatmaps* in E and F; note: epidermal β-gal expression in control (E) is due to presence of *lacZ* balancer; Alk protein is observed in *Alk*^*1*^*/Alk*^*10*^ animals (F) as these alleles encode non-functional Alk protein truncations detected with anti-Alk). **(G)** Ectopic expression of the Alk ligand Jeb, leads to activation of Alk signaling in all cells of the VM (*arrowhead*) and is marked by expression of Org-1 in *blue* resulting in increased *lacZ* expression from *AlkE2*.*7* in all cells of the VM (compare β-gal *heatmaps* in E and G). Close up regions in E-G are indicated with *dashed boxes*. Scale bars: 50 μm and 10 μm (embryo and close up, respectively).

To study *Alk* expression during embryogenesis, we employed transgenic *GAL4*-lines containing overlapping DNA sequences corresponding to *Alk* 5-prime upstream regions (Fig 1A, S1 Fig), aiming to identify regulatory elements with activity in the visceral mesoderm (VM). *AlkEI6*.*5-GAL4* was previously described [1] as driving expression in the trunk VM with stronger expression in founder cells (FCs) (Fig 1B, stage 11, *arrowhead*). We also noted that the *AlkEI6*.*5-GAL4* driver was expressed in the amnioserosa (AS), in keeping with earlier observations that *Alk* mRNA is expressed in the dorsal-most region of the embryo corresponding to the presumptive AS at the early gastrulation stage (S2A and S2B Fig) [1]. We next analyzed *AlkE4-GAL4*, which contains 2.4 kb of the *AlkEI6*.*5-GAL4* region and an additional 1.6 kb upstream (4.0 kb in total). This *GAL4*-driver promotes expression in a similar pattern to *AlkEI6*.*5-GAL4*, suggesting this DNA region also contains regulatory elements involved in *Alk* transcriptional regulation (Fig 1C). In addition, *AlkE2*.*7-GAL4*, covering a shorter sequence within *AlkEI6*.*5* and *AlkE4*, displays activity in the entire trunk VM, being considerably stronger in FCs (Fig 1D, *arrowhead*).

To ensure the specificity of our transgenic lines for the *Alk* locus flanking genes we performed *in situ* hybridization on both neighboring genes namely *CG5065* (upstream) and *gprs* (downstream) (Fig 1A, S3 Fig). Neither *CG5065* nor *gprs* is expressed in a pattern similar to that of *Alk* in the VM, suggesting that any VM expressing region identified flanking the *Alk* locus may be involved in the regulation of *Alk* transcription.

The elevated level of expression of *AlkEI6*.*5-GAL4* and *AlkE2*.*7-GAL4* in FCs compared with other cells of the developing VM suggests *Alk* may respond to its own signaling. Since signaling in the FCs is driven by activation of Alk by its ligand Jelly Belly (Jeb), we examined expression of *AlkE2*.*7-GAL4* in either the absence of Alk activity (*Alk*^*1*^*/Alk*^*10*^), or upon activation of Alk by overexpression of Jeb in the VM. *AlkE2*.*7-GAL4* expression in the FCs was reduced in *Alk*^*1*^*/Alk*^*10*^ mutants (Fig 1F; *arrowhead*). In contrast, overexpression of Jeb resulted in robust expression of *AlkE2*.*7-GAL4* in all cells of the VM (Fig 1G; *arrowhead*). These results suggest that *Alk* expression in the VM is positively regulated by Alk signaling, representing a positive feedback loop. Thus, we have identified CRMs in the 5’ region of the *Alk* locus that promote *Alk* expression in the presumptive amnioserosa and developing VM. Additionally, our preliminary *GAL4* analysis suggests the presence of inhibitory modules within this region that likely contribute to the overall regulation of *Alk* expression.

### Analysis of *Alk* enhancer regions *in vivo* by CRISPR/Cas9-mediated deletion identifies a critical role for the *Alk-RB* promoter

**Fig 2:**
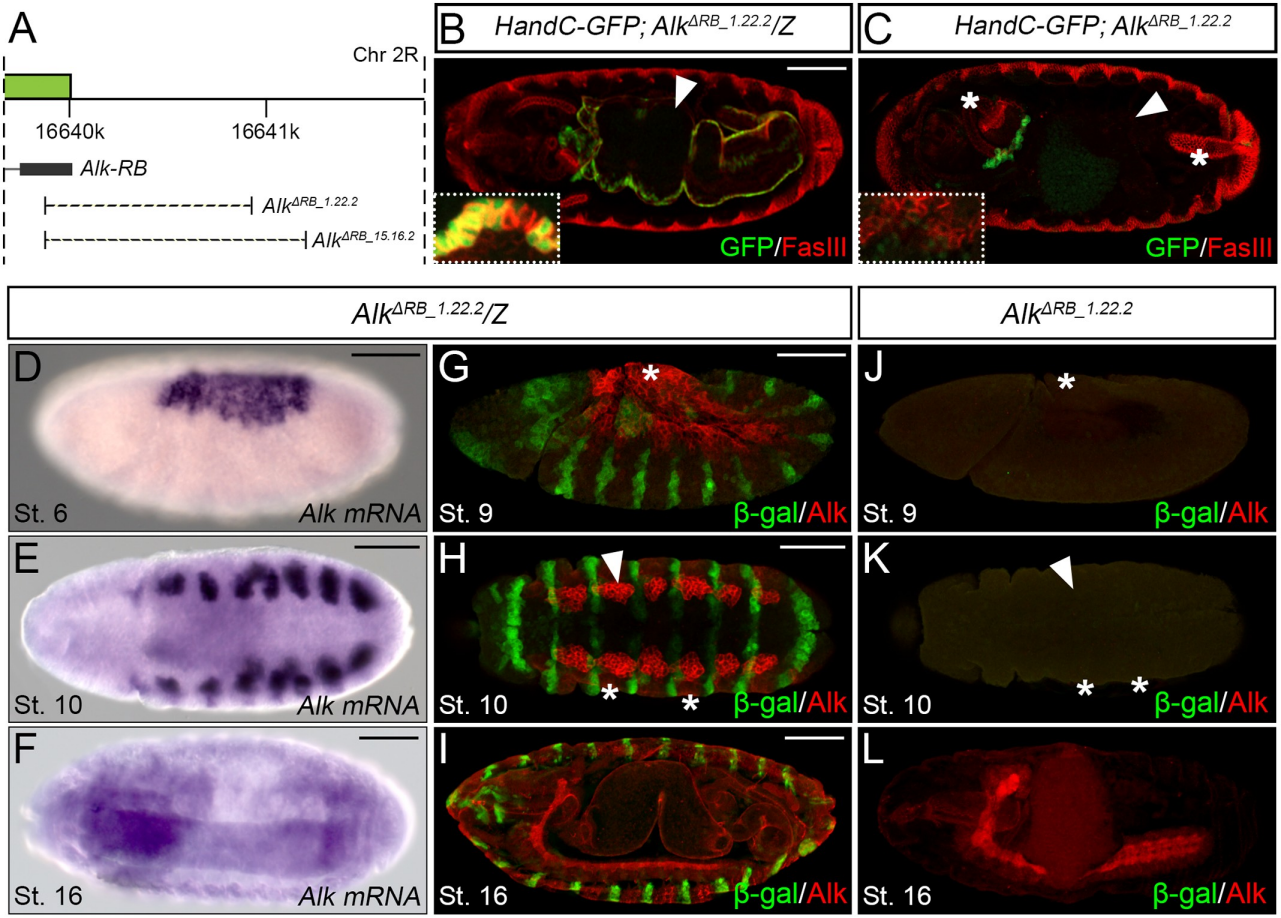
Functional analysis of *Alk-RB* by CRISPR/Cas9 mediated deletion. **(A)** Schematic overview indicating the two independent deletions generated to disrupt the *Alk-RB* promoter (*dashed lines*), referred as *Alk*^*ΔRB_1*.*22*.*2*^ and *Alk*^*ΔRB_15*.*16*.*2*^. The *Alk* locus is shown in *green* and the first exon of *Alk-RB* in *black*. **(B)** FasIII staining of the chambered midgut (*arrowhead*) of *Alk*^*ΔRB_1*.*22*.*2*^ heterozygotes in stage 16 wild-type embryos. Close up of a stage 11 *Alk*^*ΔRB_1.22.2*^ heterozygote embryo showing *HandC-GFP* reporter as a marker for FC specification in the VM. **(C)** *Alk*^*ΔRB1*.*22*.*2*^ mutants fail to specify FCs from VM precursors FCs (inset, close up of a stage 11 *Alk*^*ΔRB1*.*22*.*2*^ mutant embryo), resulting in stage 16 embryos lacking a FasIII positive midgut (*arrowhead*), phenocopying *Alk* null mutants. FasIII is still present in the foregut and hindgut (*asterisks*). **(D-F)** *Alk* mRNA is expressed in the presumptive AS (D, stage 6), the VM (E, stage 10) and the CNS (F, stage 16). **(G-L)** *Alk*^*ΔRB1*.*22*.*2*^ mutant embryos lack Alk protein in the presumptive AS (J, stage 9, *asterisk*; compare with G), the VM (K, stage 10, *arrowhead*, compare with H) and the epidermis (K, stage 10, *asterisks*, compare with H). In contrast to the lack of *Alk* mRNA and protein in the AS, VM and epidermis, *Alk*^*ΔRB1*.*22*.*2*^ embryos display normal Alk protein levels in the CNS (stage 16, compare L with I). Scale bars: 50 μm.

ChIP experiments performed by the Furlong laboratory have identified a 547 bp CRM (*MesoCRM-880*) overlapping the *AlkE2*.*7* fragment that binds Bin, Bap, Mef, Tin and Twi TFs [16] (shown schematically in Fig 1A, S1 Fig). Later analysis by the Frasch group identified a 1,984 bp region (*AlkE301*) in a genome wide Tin ChIP analysis that drives expression in the VM [17] (shown schematically in Fig 1A, S1 Fig). Together with our *GAL4* analyses these results suggest that the *Alk-RB* promoter may be important for the VM expression of Alk. To functionally address the role of *Alk-RB* we generated deletion mutants targeting the *Alk-RB* isoform with CRISPR/Cas9 [19–21], employing two independent single guide RNA (*sgRNA*) combinations. This resulted in genomic deletions of 1053 bp (represented by *Alk*^*ΔRB_1*.*22*.*2*^) or 1325 bp (represented by *Alk*^*ΔRB_15*.*16*.*2*^) in the region of the *Alk-RB* 5’UTR (Figs 1A and 2A; S1 Fig; S1 Table). Both homozygous mutants were embryonic lethal. We further examined the visceral morphology of homozygous *Alk*^*ΔRB_1*.*22*.*2*^ mutant embryos and control siblings using Fasciclin III (FasIII) as marker for differentiated VM. In control embryos FasIII was expressed in the visceral musculature surrounding the entire midgut, which at later stages of embryogenesis is subdivided into four chambers (Fig 2B; *arrowhead*). In *Alk*^*ΔRB_1*.*22*.*2*^ embryos, FasIII-positive midgut muscles were absent while FasIII-expression could still be detected in the embryonic foregut and hindgut respectively (Fig 2C), resembling the *Alk* mutant phenotype [2]. In agreement with their mutant phenotype, *Alk*^*ΔRB_1*.*22*.*2*^ mutants lacked detectable Alk mRNA and protein in the VM (Fig 2K, S4 Fig) compared to wild-type animals (Fig 2E and 2H, S4 Fig), while *Alk* expression levels in the CNS were similar to those observed in control embryos (Fig 2I and 2L; S4 Fig). *Alk* expression was also lost in the AS and epidermis of *Alk*^*ΔRB_1*.*22*.*2*^ mutants (Fig 2J and 2K, *asterisks*). Therefore, expression from the *Alk-RB* promotor drives *Alk* expression in the embryonic VM, AS and epidermis and is critical for proper formation of the midgut musculature.

### Identification of potential regulators of *Alk* expression by high throughput yeast one-hybrid screening

A 3.6 kb genomic region that covered the putative VM and epidermal *Alk* enhancer regions identified in our initial experiments (Fig 1A) was subjected in parallel to high throughput yeast one-hybrid (Y1H) and more detailed reporter gene analyses. Six fragments (denoted *AlkEB6* –*AlkEB11*; S1A Fig; S2 Table) of approximately 700 bp in length, including a ~100 bp overlap between neighboring fragments, were analyzed.

**Fig 3:**
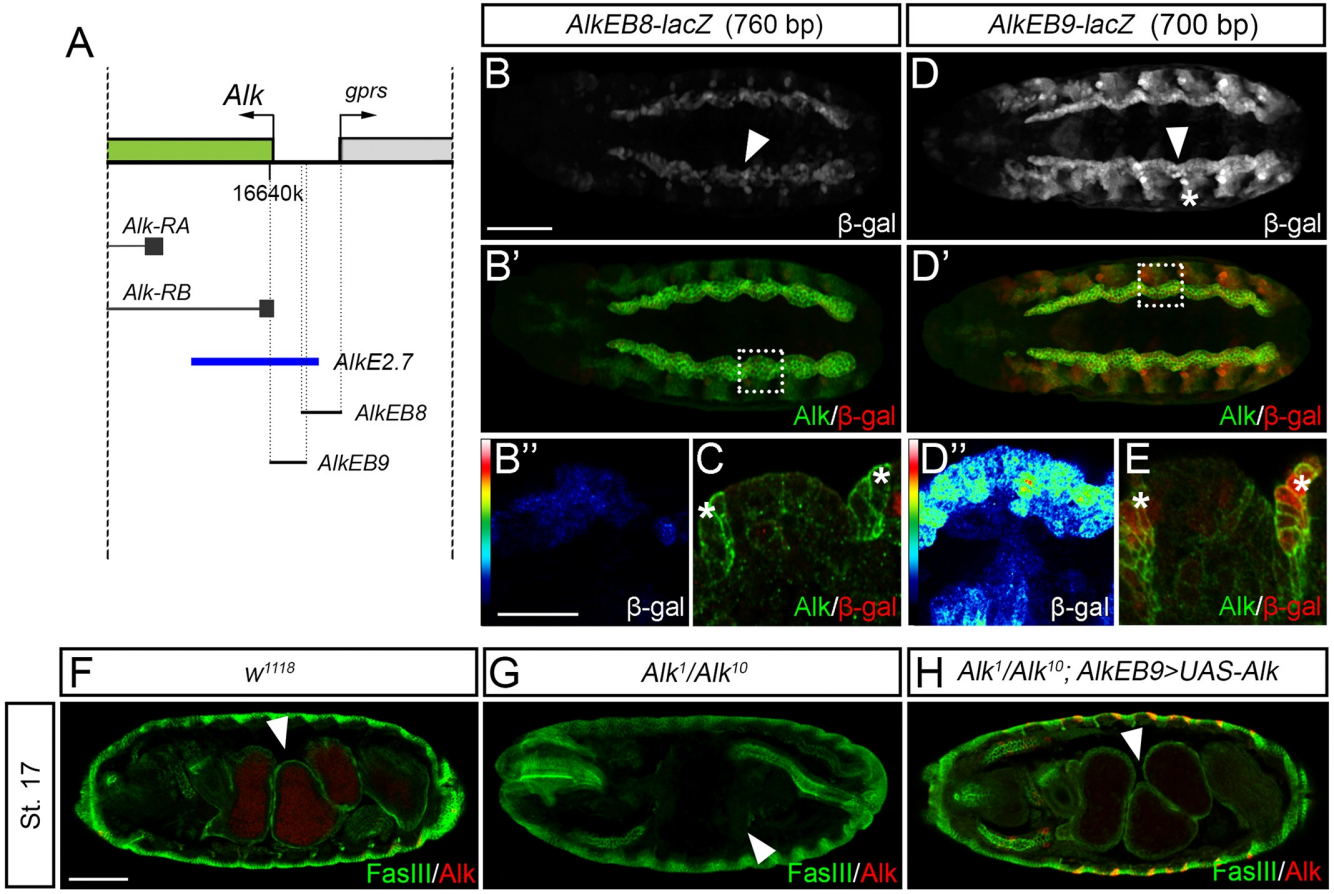
*In vivo* characterization of *Alk* locus five prime regulatory regions. **(A)** Schematic representation of the 5’ region of the *Alk* locus together with the regions covered by transgenes employed for reporter activity analysis, namely *AlkEB8* and *AlkEB9* (*black lines*). *AlkE2*.*7* is shown for comparison (*blue*). **(B, D)** *AlkEB8-lacZ* and *AlkEB9-lacZ* transgenic reporter flies showed VM activity (*arrowheads*, *dashed boxes* in B’ and D’ indicate close ups in B” and D”), with stronger expression of *AlkEB9-lacZ* (compare B, B” and D, D”; quantified in S5 Fig). *AlkEB9-lacZ* also displays reporter activity in the epidermis (E compare with C, stage 14; *asterisks*). **(F-H)** The gut phenotype of *Alk* loss of function mutants (G, *arrowhead*) can be rescued by ectopic expression of Alk driven by *AlkEB9-GAL4* (H), indicating that *AlkEB9* contains sufficient regulatory information to drive expression in the VM during embryogenesis. Scale bars: 50 μm and 10 μm (embryo and close up, respectively).

Embryonic *lacZ* reporter activity was observed with only two of the DNA fragments studied, namely *AlkEB8* and *AlkEB9* (Fig 3A–3E). *AlkEB8* displayed weaker activity in the VM than that observed with *AlkEB9* (Fig 3B, 3B”, 3D and 3D”, *arrowheads*; quantified in S5 Fig). In addition to VM expression, *AlkEB9* was also expressed in the AS and epidermis where it overlapped with Alk protein (Fig 3E, *asterisks*; S2D Fig). No expression in the AS and epidermis was observed in *AlkEB8* (Fig 3C, *asterisks*; S2C Fig). To further confirm that *AlkEB9* contains important enhancer elements for *Alk*, we performed rescue experiments using *AlkE9-GAL4* (Fig 3F–3H). Ectopic over-expression of Alk (*AlkEB9-GAL4>UAS-Alk*) in an *Alk*^*1*^*/Alk*^*10*^ mutant background resulted in a rescue of the embryonic gut phenotype (Fig 3H). Therefore, the *AlkEB9* genomic region contains sufficient regulatory information to allow rescue of the embryonic *Alk* VM expression.

**Fig 4:**
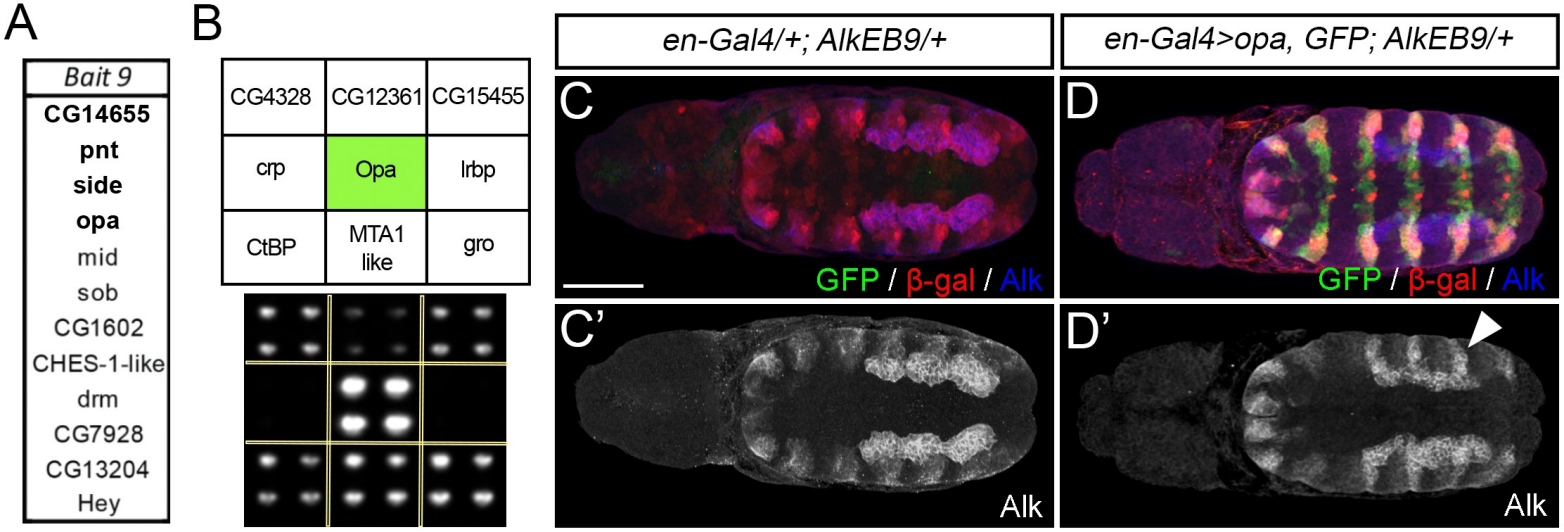
Identification of TFs binding to *AlkEB9* by high throughput yeast one-hybrid screening. **(A)** TFs identified as binding to DNA *Bait 9* (*AlkEB9*) by Y1H screening. Highlighted in **bold** are TFs bound to *Bait 9* in all replicates performed. **(B)** Image analysis of a representative Y1H screen plate for DNA *AlkEB9* (*Bait 9*), highlighting the interaction with Opa (shown in *green*). Additional quadrants of colonies shown represent negative average background growth. **(C, C’)** Expression of the *AlkEB9-lacZ* element and Alk protein in the VM and epidermis at stage 11. **(D, D’)** Ectopic expression of Opa under *en-GAL4* control leads to an expansion of both Alk protein (*arrowhead*) and *AlkEB9-lacZ* reporter gene expression (in *red*) in the embryonic epidermis at stage 11. Scale bar: 50 μm.

High throughput Y1H was carried out on the same six fragments employing a library of *Drosophila* TFs fused to the yeast *GAL4* activation domain [18] (Fig 1A, S1 Fig). Based on our reporter gene analysis we focused on the *AlkEB9* DNA bait Y1H data set aiming to functionally characterize novel transcriptional regulators of *Alk*. A set of TFs was identified to bind to the *AlkEB9* DNA bait by Y1H screening (Fig 4A). Among these, Odd-paired (Opa) (Fig 4B), Pointed (Pnt), Side and CG14655 bound to the *AlkEB9* DNA bait and promoted growth in selective media in all biological replicates performed. We further investigated a role for TFs binding *AlkEB9* in Alk transcriptional regulation *in vivo*, employing *paired (prd)-GAL4*, which drives expression in alternating parasegments and offers internal control of *Alk* expression levels in the epidermis. In this assay both Opa and Pnt were identified as potential regulators of *Alk*, with Opa inducing and Pnt repressing *Alk* expression (S6 Fig). Of the TFs tested in this study, Opa was the only one that resulted in an increase in Alk protein. We also overexpressed *opa* with the *engrailed (en)-GAL4* driver which resulted in an increase in *AlkEB9-lacZ* reporter activity as well as Alk protein levels in the epidermis (Fig 4C–4D’), indicating that Opa is sufficient to promote *Alk* expression. Therefore we focused on a more detailed investigation of the role of Opa *Alk* transcriptional regulation.

### *AlkEB9* contains functional Opa binding sites

**Fig 5:**
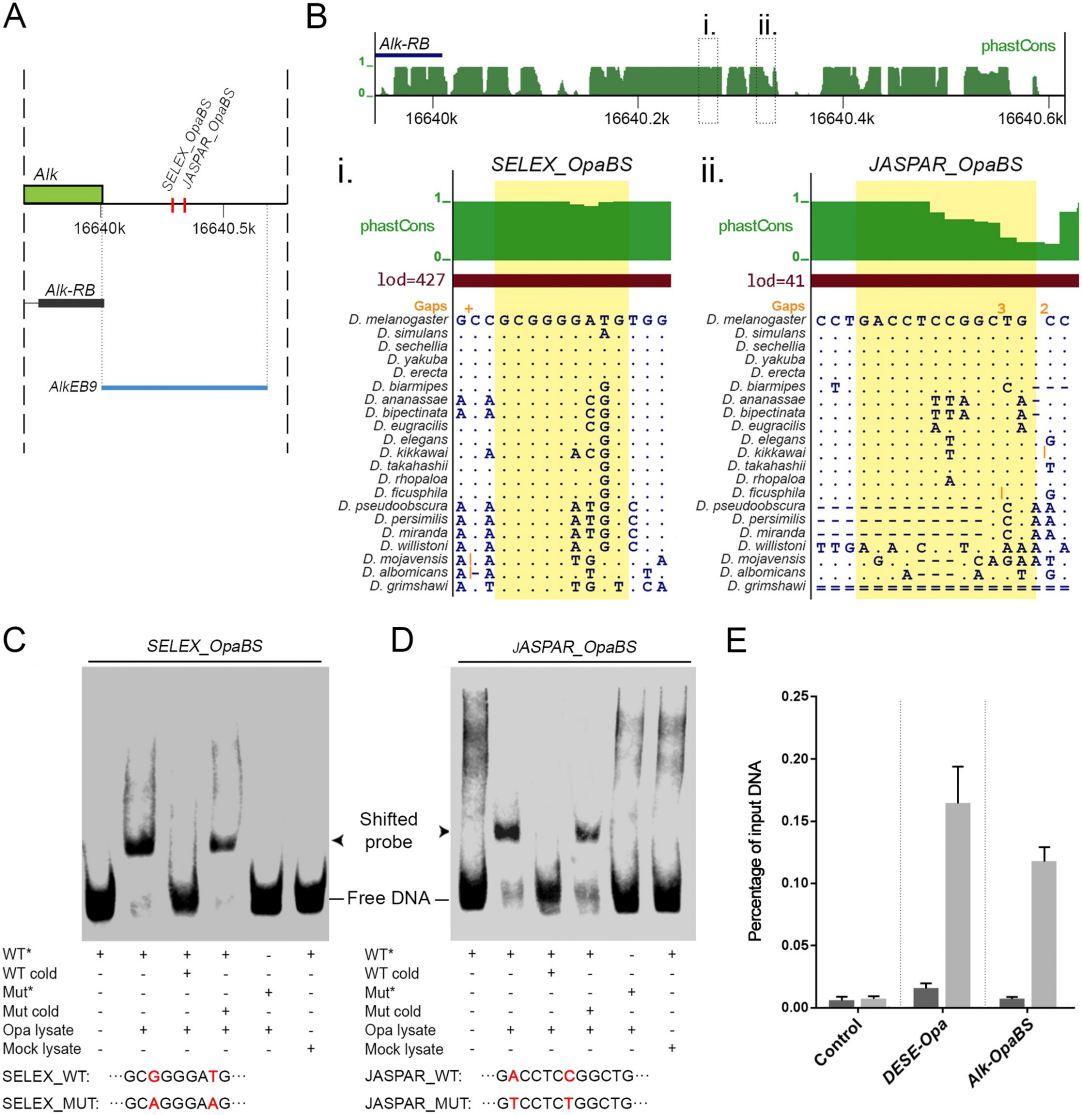
Functional validation of Opa as a putative regulator of *Alk*. **(A)** Schematic overview of *AlkEB9* element (*blue line*) and the predicted Opa binding sites, referred to as *SELEX_OpaBS* and *JASPAR_OpaBS* (in *red*). **(B)** PhastCons analysis of sequence conservation among 21 *Drosophila* species (in *green*) along *AlkEB9*, including the predicted Opa binding sites (marked with dashed boxes (i) and (ii)). Base resolution of the phastCons analysis for *SELEX_OpaBS* (i) and *JASPAR_OpaBS* sites (ii) is shown, Opa binding sites are highlighted in *yellow*. **(C, D)** Binding affinity of Opa to both *SELEX_OpaBS* and *JASPAR_OpaBS* sequences as assessed by EMSA. Opa-induced shifts could be competed by addition of non-labelled probe, but not by unlabeled mutated probes. Sequences of both wild type and mutant probes are indicated, mutated residues are depicted in *red*. **(E)** ChIP assay employing either pre-immune serum control (*dark grey bars*) or anti-Opa serum from the same rabbit (*light grey bars*) for a control intergenic region (*control*), an Opa binding region within the *slp1* enhancer (*DESE-Opa*) and a 140 bp region containing both the *SELEX_Opa-BS* and *JASPAR_OpaBS* sequences within *AlkEB9* (*Alk-OpaBS*). Enrichment of the different DNA segments in the immunoprecipitates are reported as a percentage of input DNA, with error bars representing the mean ± SD from three technical replicates of the qPCR.

Employing the JASPAR online prediction tool [22], we were able to identify a potential Opa binding site (BS) in the *AlkEB9* sequence, *JASPAR_OpaBS* (GACCTCCGGCTG) (Fig 5A and 5B). In addition, we identified another Opa BS similar to the Opa consensus motif previously reported by [23] and therefore referred to as *SELEX_OpaBS* (GCGGGGATG) (Fig 5A and 5B). Employing the phastCons database, which identifies evolutionarily conserved elements in a multiple alignment, to analyze this sequence, we found that both binding sites are conserved among *Drosophila* species (Fig 5B; conservation score in *green*; *Opa BS* highlighted in *yellow*) [24, 25]. We next assessed the ability of Opa to specifically bind these predicted sites by electrophoresis mobility shift assay (EMSA). EMSA was performed on the *SELEX_OpaBS* and *JASPAR_OpaBS* sequences, incubating probes with cell lysates from Opa-expressing HEK293 cells in the presence of poly(dI-dC) to prevent non-specific binding. Addition of Opa lysate to the binding reaction resulted in a shift of both *SELEX_OpaBS* and *JASPAR_OpaBS* probes and was reversed by addition of 100 fold molar excess of non-labelled probe (Fig 5C and 5D). In contrast, addition of cold probes that were mutated within the *SELEX* and *JASPAR* binding sites, based on published data [23], was unable to compete the shift generated upon addition of Opa to the labelled wild-type probe. Furthermore, labelled mutated *SELEX_OpaBS* and *JASPAR_OpaBS* probes did not exhibit a mobility shift upon incubation with Opa (Fig 5C and 5D). The above observations led us to characterize the interactions of the Opa with the *Alk* locus by chromatin immunoprecipitation (ChIP). Consistent with Y1H and EMSA analyses, Opa association is detected with a region upstream of the *Alk* promoter that spans both the SELEX_OpaBS and JASPAR_OpaBS sequences in chromatin from wild-type embryos (Fig 5E).

**Fig 6:**
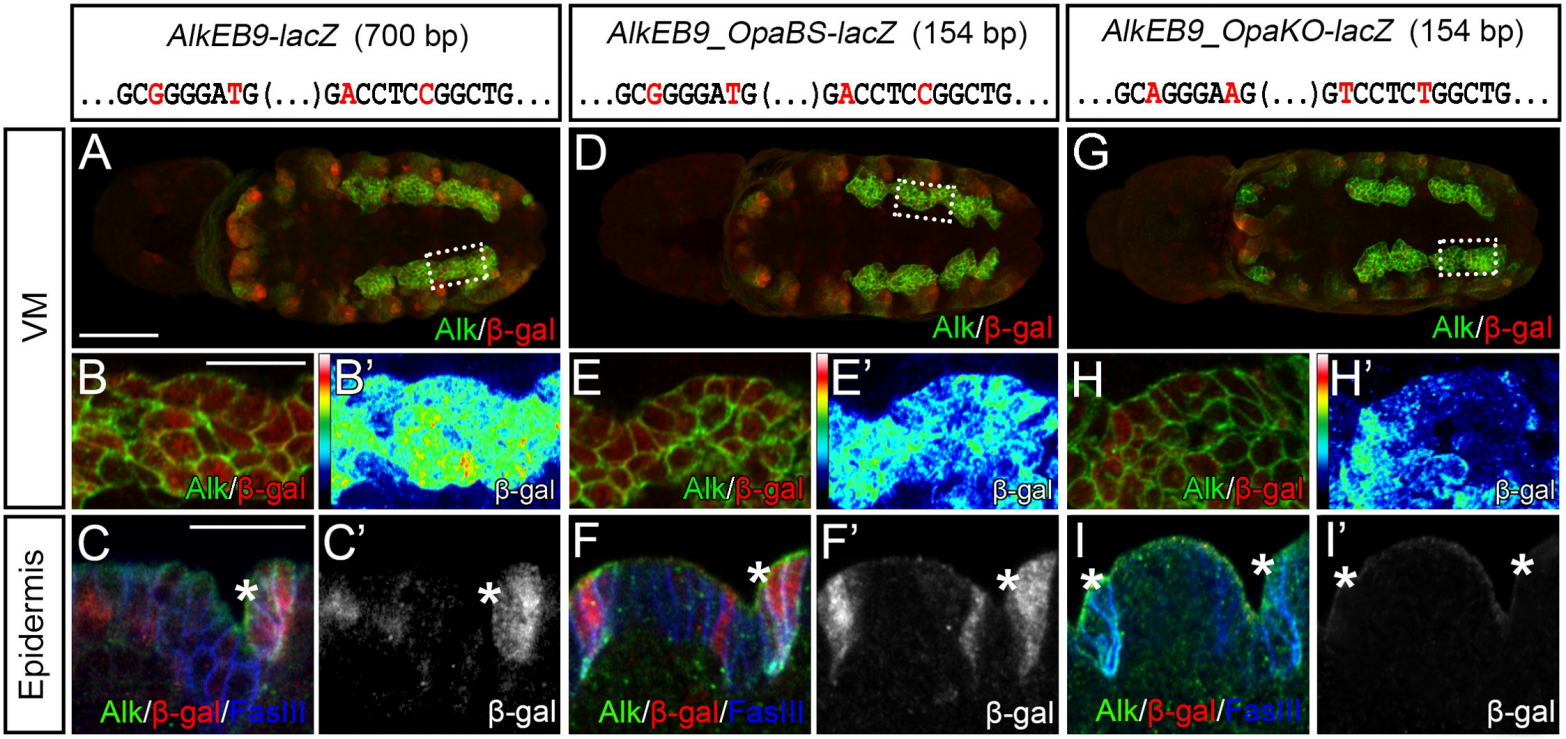
*In vivo* Opa binding sites analysis in the *AlkEB9* element. **(A-C’)** *AlkEB9* drives *lacZ* reporter expression in the VM (stage 11, *dashed box* in A indicates area of close ups in B, B’) and epidermis (stage 14, *asterisk*). **(D-F’)** The 154 bp *AlkEB9_OpaBS-lacZ* reporter drives expression in a pattern similar to that of *AlkEB9-lacZ*, and is slightly weaker in the VM when compared to *AlkEB9* (stage 11, compare B’ and E’, *dashed box* in D indicates area of close ups in E, E’) but intact in the epidermis (F, F’, compare with C, C’; stage 14; *asterisks*). The wild-type *SELEX_OpaBS* and *JASPAR_OpaBS* sequences are shown for *AlkEB9-lacZ* and *AlkEB9_OpaBS-lacZ* reporters with the mutated nucleotides in the *AlkEB9_OpaKO-lacZ* indicated in *red*. **(G-I’)** Mutation of the Opa binding sites within *AlkEB9_OpaBS* (*AlkEB9_OpaKO-lacZ*) decreases *lacZ* reporter activity in the VM (stage 11, compare E’ and H’, *dashed box* in G indicates area of close ups in H, H’), but abolishes it in the epidermis (I, I’, compare with F, F’; stage 14; *asterisks*). Scale bars: 50 μm and 10 μm (embryo and close ups, respectively). Quantification of *lacZ* activity in S7 Fig.

To address the importance of the *JASPAR_* and *SELEX_OpaBS* for *in vivo* *Alk* transcription we first attempted to identify a minimal region within the *AlkEB9* region that could drive VM expression. This analysis led to the identification of a 154 bp fragment including both *SELEX* and *JASPAR* Opa binding sites (*AlkEB9_OpaBS*; schematically shown in S1 Fig) that drives strong VM and epidermal expression, similar to that observed with the 700 bp *AlkEB9* fragment (Fig 6A–6F’). Quantification revealed that VM expression from *AlkEB9_OpaBS* was weaker than that of the 700 bp *AlkEB9-lacZ* reporter (Fig 6B’ and 6E’; S7 Fig), while expression in the epidermis appeared similar in both the 154 bp and 700 bp fragments (Fig 6C’ and 6F’; S7 Fig). In order to examine the role of the predicted Opa binding sites, we introduced the same mutations as in our earlier EMSA analysis within the 154 bp *AlkEB9_OpaBS* minimal region to create *AlkEB9_OpaKO-lacZ*. Mutation of these binding sites led to a loss of *lacZ* expression in both the VM and epidermis (Fig 6G–6I’, quantified in S7 Fig), implying that the predicted Opa binding sites in *AlkEB9* indeed contribute to expression from this element. While mutation of Opa binding sites led to a significant reduction of reporter gene expression in the VM (Fig 6H’; quantified in S7 Fig) this was not complete, in contrast to a complete loss of detectable *lacZ* activity in the epidermis (Fig 6I’; quantified in S7 Fig). Taken together, these data show that the *AlkEB9* genomic region contains sequence-specific binding sites for Opa that regulate expression from *Alk* enhancer elements.

### Opa is required for tissue specific *Alk* expression during embryogenesis

To further dissect the potential role of Opa as a regulator of *Alk* expression, we examined *opa* expression during embryogenesis [26]. *opa* mRNA can be detected at stage 5 in the ectoderm and mesoderm progenitors spanning the presumptive segmented region of the embryo. At stage 9 *opa* expression decreases slightly and appears in the neuroectoderm persisting until late embryo stages. In the VM, *opa* mRNA is observed in a dynamic pattern, where it is expressed in a clustered fashion in PS 3–5 and PS 9–12 (S8 Fig).

**Fig 7:**
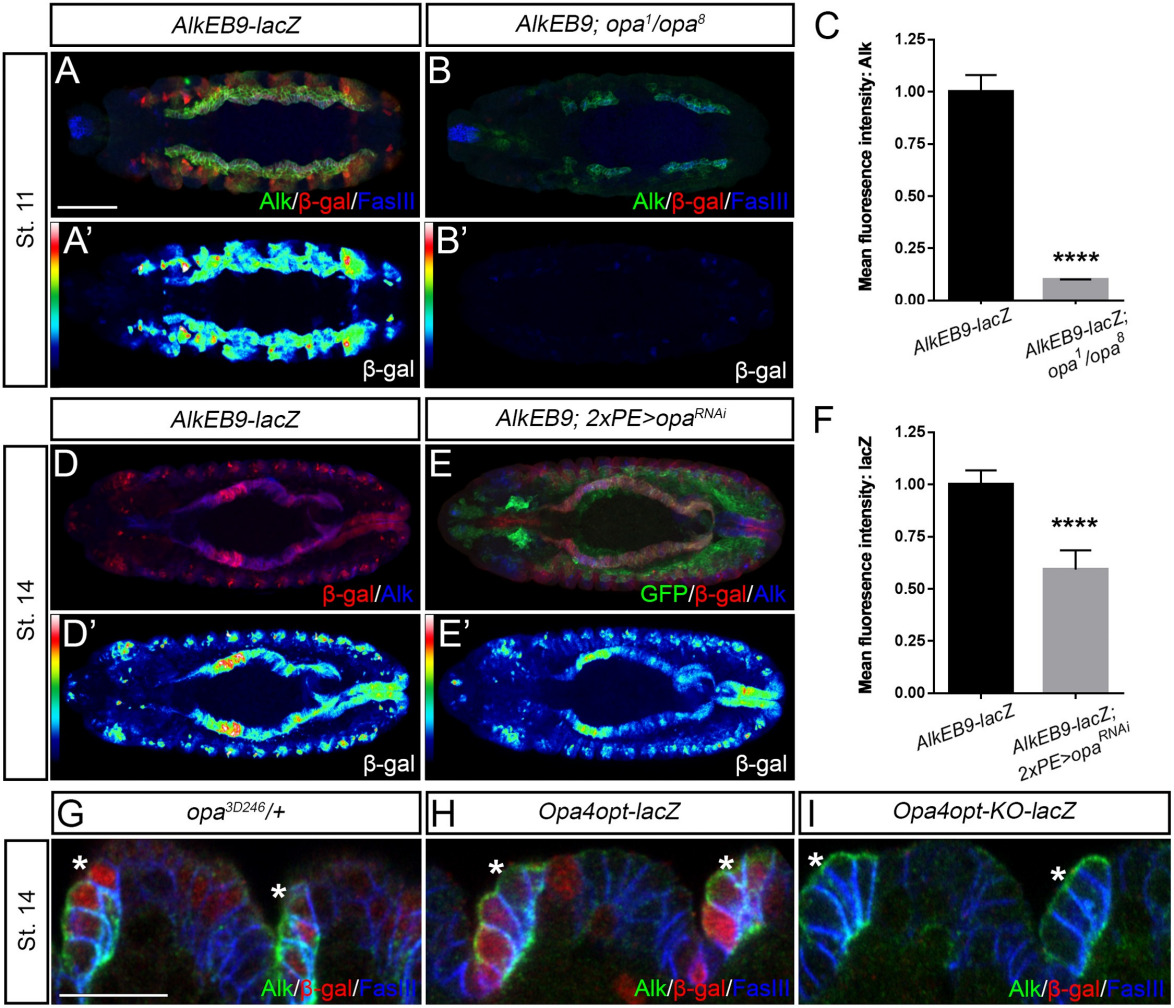
Contribution of Opa to Alk expression during embryogenesis. **(A)** Expression of *AlkEB9-lacZ* (*red*) in the VM at stage 11 reflects Alk protein expression (*green*). **(B)** Loss of expression of *AlkEB9-lacZ* in the VM of *opa*^*1*^*/opa*^*8*^ mutants (*red*; compare *heatmaps* in A’ and B’) along with reduced Alk protein levels in the VM and complete abrogation in epidermis (B). **(C)** Quantification of Alk protein levels in the VM shows a significant decrease in *opa*^*1*^*/opa*^*8*^ animals (n = 10 animals, p<0.0001). **(D-F)** Ectopic expression of *opa*^*RNAi*^ with *2xPE-GAL4* as driver decreases *AlkEB9-lacZ* reporter expression (compare D’ and E’; quantified in F; n = 10 animals, p<0.0001). **(G)** Expression of the *opa*^*3D246*^ enhancer trap in Alk-positive cells of the embryonic epidermis (*asterisks*). **(H)** The *Opa4opt-lacZ* reporter, containing Opa binding sites (*SELEX_OpaBS*), is active in epidermal cells expressing Alk protein (*asterisks*). **(I)** Mutation of the *SELEX_OpaBS* binding sites (*Opa4opt-KO-lacZ*) results in loss of reporter gene expression in epidermal cells expressing Alk protein (*asterisks*). Scale bars: 50 μm (A-B’, D-E’) and 10 μm (G-I).

We next examined the reporter expression of *AlkEB9-lacZ* in *opa* loss-of-function mutants (*opa*^*1*^*/opa*^*8*^). While *AlkEB9-lacZ* is activated in the entire VM and epidermis in wild-type embryos (Fig 7A and 7A’), *opa*^*1*^*/opa*^*8*^ mutant embryos display only weak reporter activity during embryogenesis (Fig 7B and 7B’; quantified in Fig 7C). The severe developmental defects observed in *opa*^*1*^*/opa*^*8*^ mutants make analysis difficult, however we noted lower levels of Alk protein in the VM and a complete loss of detectable Alk in the epidermis of *opa* mutant animals, in agreement with the loss of *AlkEB9-lacZ* activity (Fig 7B). These observations were supported by analysis of *RNAi*-induced Opa knockdown in the developing mesoderm employing *2xPE-GAL4* (Fig 7D–7F). We observed that embryos expressing *opa RNAi* (*2xPE-GAL4>UAS-opa*^*RNAi*^) displayed a reduction of *AlkEB9-lacZ* in the VM at later stages when compared with controls (Fig 7E’, quantified in Fig 7F).

Since Opa has been reported to be required for proper midgut formation, with *opa* mutants exhibiting an interrupted VM that fails to form midgut constrictions during embryogenesis [26], we also examined Alk signaling in the VM of *opa* mutants. *opa*^*1*^*/opa*^*8*^ mutants, examined with the FC-marker Org-1, exhibited Org-1 positive VM FCs, however, the level of Org-1 protein observed was less than in control embryos (S8B and S8C Fig). Since reductions in both Alk and Org-1 protein were seen in *opa*^*1*^*/opa*^*8*^ mutants, we asked whether Opa overexpression was sufficient to drive Alk signaling. As expected, *bap3-GAL4* driven expression of Jeb in the VM resulted in an increased expression of the *HandC-GFP* FC marker reflecting activation of Alk signaling (S8D–S8D” Fig). In contrast, *bap3-GAL4* driven expression of Opa did not increase *HandC-GFP* levels (S8E–S8E” Fig). Thus, while Alk signaling may be reduced in *opa* mutants, Opa is not sufficient to influence FC specification driven by Alk signaling in the embryonic VM.

As a complement to our analysis of *opa* mutants, we employed the *Opa4opt-lacZ* transgene as readout for Opa activity, focusing on the epidermis. *Opa4opt-lacZ* contains four tandem copies of the *SELEX* determined *Opa-BS* [23]. In parallel we analyzed the *opa*^*3D246*^ *lacZ* enhancer trap which reflects *opa* expression [26]. We observed expression of both *opa*^*3D246*^ and *Opa4opt-lacZ* in the embryonic epidermis, coinciding with Alk protein (Fig 7G and 7H), suggesting that Opa is both expressed and active in these cells. Furthermore, a mutant *Opa4opt-lacZ* transgene, called *Opa4opt-KO-lacZ*, in which the Opa binding sites are mutated, no longer displayed expression overlapping with Alk in the embryonic epidermis (Fig 7I).

Taken together, this data supports an important role for Opa in driving embryonic *Alk* transcription, particularly in the epidermis, through the *AlkEB9* regulatory region. However, in agreement with our earlier analyses, *Alk* expression in the VM does not depend only on Opa activity, since Alk protein is still observed in the VM of *opa*^*1*^*/opa*^*8*^ loss of function animals (Fig 7B).

### Opa binding sites in the *Alk* enhancer region regulate Alk protein expression in a tissue-specific context

**Fig 8:**
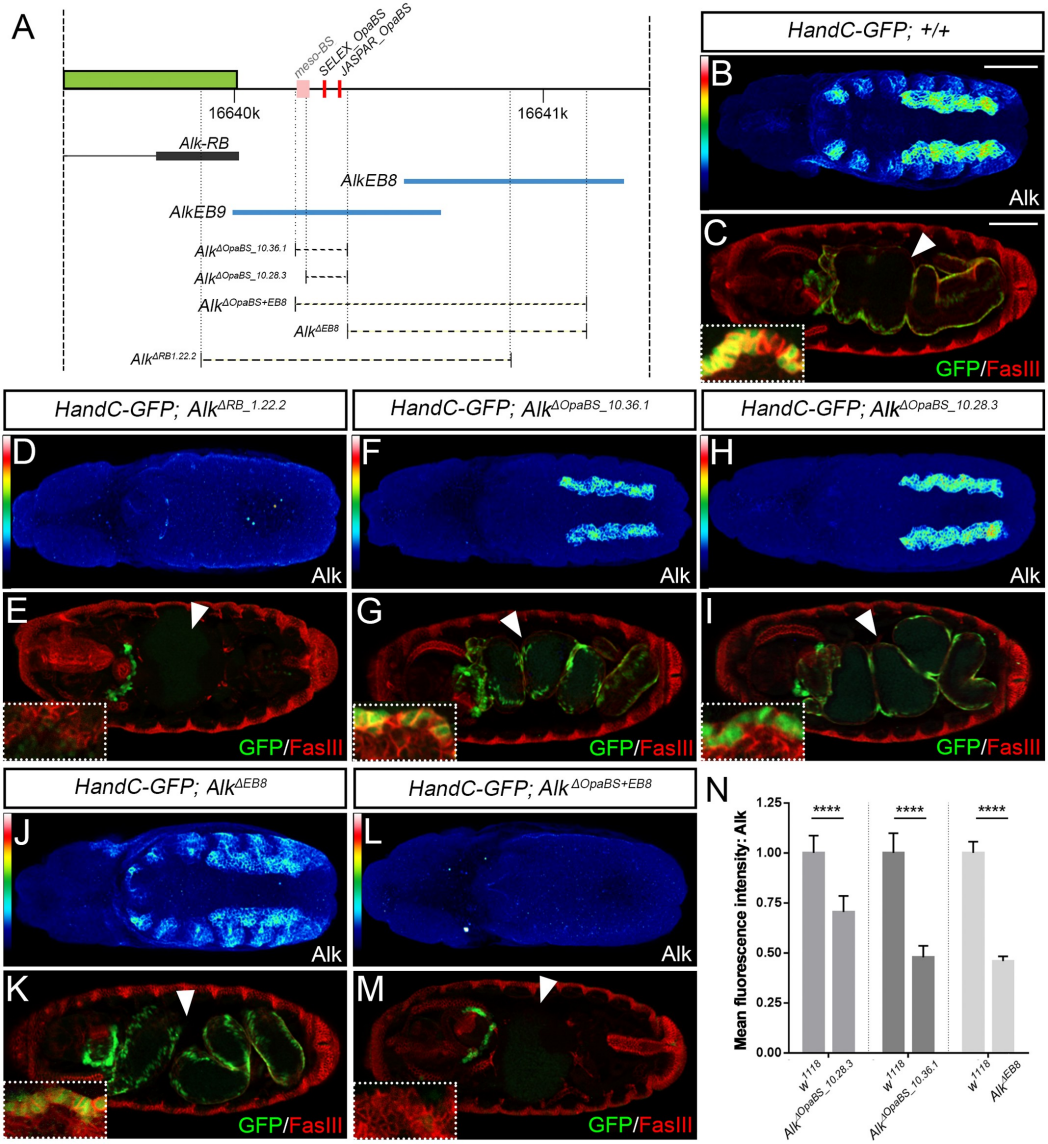
The Opa binding site containing CRM is crucial for tissue-specific *Alk* expression during embryogenesis. **(A)** Overview of the CRISPR/Cas9 deletions generated (*dashed lines*) within the *AlkEB9* and *AlkEB8* enhancer region of the *Alk* locus (*blue lines*). Predicted binding sites shown in *red*. **(B, C)** Alk protein is normally expressed in the VM and adjacent epidermis of wild-type embryos at stage 11. Alk activation drives expression of the *HandC-GFP* reporter in FCs of wild-type embryos (C, inset, stage 11 embryo, *green*), resulting in midgut formation (C, stage 16, *arrowhead*). **(D, E)** *Alk*^*ΔRB1*.*22*.*2*^ mutants are indistinguishable from *Alk* null alleles in the VM, exhibiting loss Alk protein and FC specification (E inset, stage 11 *Alk*^*ΔRB1*.*22*.*2*^ mutant embryo) and lack of midgut formation (E, stage 16, *arrowhead*). **(F-I)** Both *Alk*^*ΔOpaBS_10*.*36*.*1*^ and *Alk*^*ΔOpaBS_10*.*28*.*3*^ mutants lack Alk protein in epidermis and display reduced levels of Alk protein in the VM (F, H, stage 11; quantified in N), although Alk levels in the VM are sufficient to drive FC specification (G, I, insets represent stage 11 *Alk*^*ΔOpaBS_10*.*36*.*1*^ and *Alk*^*ΔOpaBS_10*.*28*.*3*^ mutant embryos). **(J, K)** *Alk*^*ΔEB8*^ mutants show reduced Alk protein levels in the VM (J, stage 11; quantified in N), while epidermal expression of Alk appears to be unaffected (J, stage 11). Neither FC specification (K, inset, stage 11 *Alk*^*ΔEB8*^ mutant embryo) nor midgut formation (K, stage 16, *arrowhead*) are impaired in *Alk*^*ΔEB8*^ mutants. **(L, M)** *Alk*^*ΔOpaBS+EB8*^ behaves as an *Alk* null allele in the VM, lacking detectable Alk protein in the VM and epidermis (L, stage 11), and failing to specify FCs (M, inset, stage 11 *Alk*^*ΔOpaBS+EB8*^ mutant embryo) or develop a midgut (M, stage 16, *arrowhead*). **(N)** *Alk*^*ΔOpaBS_10*.*36*.*1*^, *Alk*^*ΔOpaBS_10*.*28*.*3*^ and *Alk*^*ΔEB8*^ mutants show a significant decrease in Alk protein in the VM when compared to control embryos (n = 10 animals per genotype, **** p≤0.0001). Scale bars: 50 μm.

Given the presence of Opa binding sites proximal to the *Alk-RB* isoform promoter, together with the loss of reporter gene activity after deletion of these sites, we next addressed their *in vivo* relevance for *Alk* transcriptional regulation. CRISPR/Cas9 genome editing was again employed to delete the identified Opa binding sites (*Opa-BS*) in the *AlkEB9* enhancer region of the *Alk* locus (Fig 8A; S1 Fig). This resulted in isolation of two viable *Alk*^*ΔOpaBS*^ mutants: *Alk*^*ΔOpaBS_10*.*28*.*3*^ and *Alk*^*ΔOpaBS_10*.*36*.*1*^ (Fig 8A and 8F–8I; S1 Fig). Loss of 151 bp containing the Opa binding sites in *Alk*^*ΔOpaBS_10*.*28*.*3*^ mutants led to a complete loss of detectable Alk protein in the amnioserosa and epidermis (Fig 8H; S1 Table), indicating this region is essential for *Alk* expression in these tissues. We also observed reduced Alk protein levels in the VM when compared to control embryos at the same stage (Fig 8H, compare with Fig 8B; quantified in Fig 8N). In close proximity to the Opa binding sites we also observed a cluster of highly scoring JASPAR-predicted binding sites for mesodermal TFs (Bap, Sna and Tin) in the *AlkEB9* genomic region, here designated as *meso-BS* (Fig 8A; S1 Fig). Deletion of this *meso-BS* region alone, in *Alk*^*ΔmesoBS*^ embryos, does not appear to affect either Alk protein levels or the formation of a fully developed gut (S1 and S9 Figs; S1 Table). Interestingly, *Alk*^*ΔOpaBS_10*.*36*.*1*^ removes 178 bp including both the *Opa-* and the *meso-BS* sites allowing us to functionally address the contribution of the *meso-BS* region relative to the Opa binding sites. Deletion of both the *meso-BS* and the *Opa-BS* regions (*Alk*^*ΔOpaBS_10*.*36*.*1*^) results in viable animals, albeit with reduced Alk protein levels when compared to those in control embryos (Fig 8F, S1 Table). Reduction of Alk protein levels in the VM was noticeably stronger in *Alk*^*ΔOpaBS_10*.*36*.*1*^ when compared with *Alk*^*ΔOpaBS_10*.*28*.*3*^ mutants (Fig 8F and 8H; quantified in Fig 8N). However, the reduced Alk protein levels observed in *Alk*^*ΔOpaBS_10*.*28*.*3*^ and *Alk*^*ΔOpaBS_10*.*36*.*1*^ were still sufficient to drive Jeb/Alk signaling in the VM as measured with *HandC-GFP* reporter expression (Fig 8G and 8I insets), and form a functional gut as visualized by FasIII staining (Fig 8G and 8I).

Since we detected VM expression activity in the overlapping *Alk* proximal *AlkEB8-lacZ* reporter (Fig 3B), we explored the contribution of the corresponding region in the *Alk* locus to regulation of *Alk* VM expression. To do this we employed CRISPR/Cas9 genomic editing to remove 808 bp covering part of *AlkEB9* (312 bp) and the majority of *AlkEB8* (647 bp) (represented by *Alk*^*ΔEB8*^) (Fig 8A; S1 Fig, S1 Table). These mutants were homozygous viable, with a wild-type VM morphology (Fig 8K; S1 Fig). Investigation of Alk protein levels in *Alk*^*ΔEB8*^ mutants revealed a decrease, but not complete loss, of Alk in the VM (Fig 8J; quantified in Fig 8N), suggesting that CRM(s) within the *AlkEB8* region are not essential but contribute to VM expression of *Alk*. Expression of *Alk* in the epidermis was not affected, in agreement with a sole epidermal CRM including the Opa binding sites within the *AlkEB9* region. To further exclude the possibility that an essential CRM might be located in the overlap between *AlkEB8* and *AlkEB9*, we generated a series of overlapping reporter constructs in this area S1 Fig. We did not observe any VM expression activity in this reporter series (S10 Fig), suggesting that two CRMs, one in the region of *AlkEB8* and one in *AlkEB9* function together drive VM expression of *Alk*.

To test the contribution of additional CRMs to VM expression of *Alk* we extended the *Alk*^*ΔEB8*^ deletion to include the Opa binding sites within *AlkEB9*. This deletion was denoted *Alk*^*ΔOpaBS+EB8*^ (Fig 8A, 8L and 8M; S1 Fig, S1 Table). *Alk*^*ΔOpaBS+EB8*^ mutants failed to express Alk protein in the VM, epidermis or AS (Fig 8L), and were homozygous lethal due to lack of FC specification (Fig 8M; inset), supporting our hypothesis of several independent CRMs within this area that are critical for *Alk* expression in the VM.

Taken together, our analysis identifies a CRM proximal to the *Alk-RB* isoform promotor that contains Opa binding sites as critical for *Alk* expression in the embryonic AS and epidermis. This region also contributes to *Alk* expression in the VM. Further deletion analysis reveals additional CRM(s) located within the *AlkEB8* fragment that contribute to regulation of *Alk* VM expression.

### Bin and Bap contribute to VM expression of *Alk*

**Fig 9:**
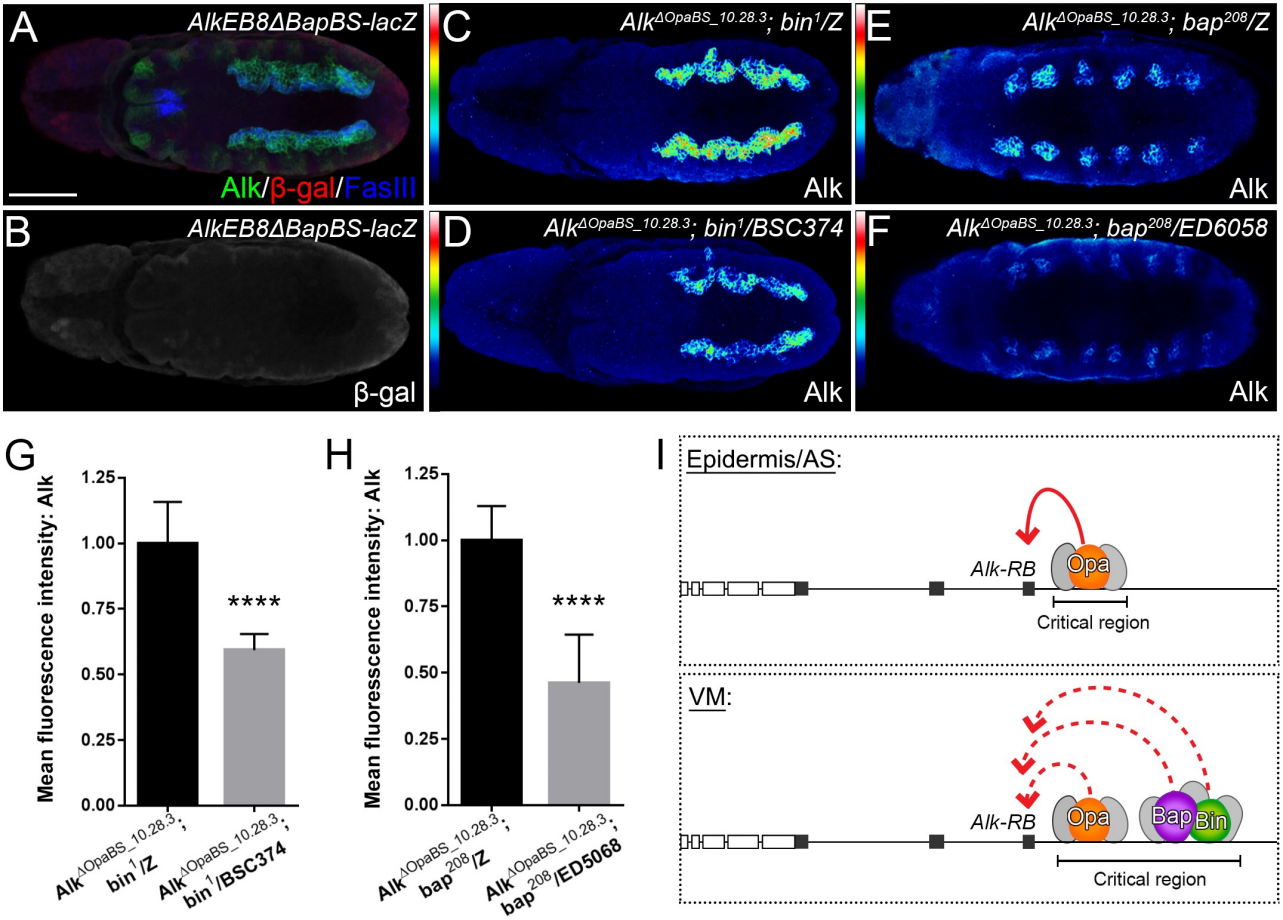
Bin and Bap contribute to regulation of *Alk* expression in the VM. **(A, B)** Removal of predicted Bap binding sites within the *AlkEB8* element (*AlkEB8ΔBapBS-lacZ*) results in loss of *lacZ* reporter activity. Alk (*green*), β–gal (*red*) and FasIII (*blue*) are detected. **(C, D, G)** Combining *Alk*^*ΔOpaBS_10*.*28*.*3*^ in a *bin* mutant background (*Alk*^*ΔOpaBS_10*.*28*.*3*^; *bin*^*1*^*/BSC374*) leads to Alk protein reduction in the VM (quantified in G; n = 10 animals, p<0.0001). **(E, F, H)** Similarly, *Alk*^*ΔOpaBS_10*.*28*.*3*^ and *bap*^*208*^*/ED6058* double mutants (*Alk*^*ΔOpaBS_10*.*28*.*3*^; *bap*^*208*^*/ED6058*) display reduced levels of Alk protein in the VM at stage 10 (quantified in H; n = 10 animals, p<0.0001). **(I)** Proposed regulatory interactions during *Alk* expression in the AS, VM and epidermis. *Alk* expression in AS and epidermis is under control of a critical CRM proximal to *Alk-RB* that binds Opa (in *orange*). Regulation may be by Opa alone, or in combination with uncharacterized additional factors (denoted in *grey*) as have been described previously by ChIP. VM expression of *Alk* involves a more extensive organization of CRMs that appear to act in a combinatorial fashion. These include the Opa binding region in *AlkEB9* also employed for the AS and epidermis, and an additional more proximal CRM(s) within the *AlkEB8* region that appears to mediate input from Bin and Bap (in *green* and *purple*, respectively) that cooperate to drive Alk expression in the VM in combination with additional factors (in *grey*). Scale bar: 50 μm.

Previous studies identified CRMs binding Bin, Bap, Twi, Tin and Mef2 in the *Alk* locus [16, 17]. In particular the ChIP and reporter gene analyses performed by Jin et al. (2013) suggested Tin binding to be important if not essential for *Alk* expression. We studied expression of Alk protein and the *AlkEB9-lacZ* reporter in *tin346/ED6058* mutant embryos (S11 Fig). Both Alk protein and reporter gene expression, could be observed in the dorsal epidermis and amnioserosa at stage 10/11 and in the epidermis at stage 14 (S11 Fig), indicating that regulation by Tin is not critical for *Alk* expression outside the VM. In contrast, *Alk* and *AlkEB9-lacZ* reporter gene expression as observed in the VM of control embryos (S11 Fig) was not observed. However, this analysis was inconclusive since it is difficult to address if, and to which extent, VM formation proceeds in *tin* mutant embryos. Bin and Bap TFs are known to have a critical function during *Drosophila* VM development [12, 15]. In our initial experiments we were unable to see any effect on *Alk* expression on ectopic expression of either Bin or Bap alone in the epidermis employing *en-GAL4* as driver (S12 Fig), however this may reflect a lack of tissue competence in our experimental approach. Therefore, we analyzed both Alk protein and *AlkEB9-lacZ* expression in *bin* and *bap* mutants focusing on VM expression. Although VM development does not proceed normally in either *bin* or *bap* mutants, we could observe Alk protein and *AlkEB9-lacZ* expression in the VM in both cases (S13B–S13C’ Fig). We next investigated *AlkEB8-lacZ* expression, which was reduced in both *bin* mutants and *bap* mutants (S13E–S13F’ Fig). On closer inspection of the *AlkEB8* region we identified four putative Bap binding sites, which we deleted to create *AlkEB8ΔBapBS-lacZ*. *AlkEB8ΔBapBS-lacZ* failed to exhibit reporter expression suggesting that Bap may be involved in *Alk* expression in the VM through binding sites within the *AlkEB8* region (Fig 9A and 9B). Based on these findings, we analyzed Alk protein levels in *Alk*^*ΔOpaBS_10*.*28*.*3*^;*bin*^*1*^*/BSC374* and *Alk*^*ΔOpaBS_10*.*28*.*3*^;*bap*^*208*^*/ED6058* double mutant backgrounds to test whether *Alk* expression was affected in a combinatorial manner. We observed a strong reduction of *Alk* expression in the VM of both *Alk*^*ΔOpaBS_10*.*28*.*3*^; *bin*^*1*^*/BSC374* mutants (Fig 9C and 9D; quantified in Fig 9G) and *Alk*^*ΔOpaBS_10*.*28*.*3*^;*bap*^*208*^*/ED6058* mutants (Fig 9E and 9F; quantified in Fig 9H), with loss of Bap appearing to have a stronger impact. These results suggest that additional factors, including Bin and Bap, contribute to regulate *Alk* expression in the VM through the *AlkEB8* region of the *Alk* locus (Fig 9I).

## Discussion

In this study we report the identification of *Alk cis*-regulatory elements and TF binding sites that control the expression of *Alk* during embryogenesis. We have been able to identify regions that regulate transcription of *Alk* in the AS, the VM and the epidermis. We further identify the Opa TF as well as Bin and Bap as regulators of *Alk* transcription in these tissues during embryogenesis. Taken together our results shed light on the regulatory mechanisms controlling *Alk* transcription and identify important *cis*-regulatory sequences required for regulation of *Alk* gene expression.

### Transcription from the *Alk-RB* promoter is essential for *Alk* expression in the visceral mesoderm

The importance of Jeb/Alk signaling *in vivo* in the embryonic VM for FC specification is well established [2–5]. From this earlier work we know that activated Alk in the VM triggers not only transcriptional activation but also post-translational modifications that promote the specification of the FC fate [3–6, 11]. In contrast, very little is known about factors that mediate *Alk* transcriptional regulation. In this study we aimed to identify CRMs and TFs important for *Alk* transcription. The *Alk-RA* and *Alk-RB* transcripts encode the same protein, but differ in their 5’ non-coding regions which employ alternative promoters [1]. This potentially allows differential expression of the *Alk-RA* and *Alk-RB* mRNA isoforms both temporally and spatially. Such regulation has been described previously for genes such as the *Drosophila* DOA kinase [27] and the BBG PDZ-protein [28], among others. Embryos in which the promoter of the *Alk-RB* isoform has been disrupted fail to express detectable Alk protein in the VM, AS and epidermis, and exhibit an *Alk* loss of function phenotype, revealing that this promoter is critical for *Alk* expression in these embryonic tissues. However, expression of *Alk* in the embryonic CNS is not compromised by the removal of the *Alk-RB* promoter and upstream sequences, suggesting that CNS expression of *Alk* is independent of the VM, AS and epidermal enhancers identified here. Taken together, our results indicate a critical requirement for *Alk-RB* expression to ensure sufficient Alk protein levels in the VM for signaling and founder cell specification, as well as for *Alk* expression in the AS and epidermis where the function of Alk is currently uncharacterized.

### Enhancer elements upstream of the *Alk* locus regulate expression in the amnioserosa, visceral mesoderm and epidermis

Previous reports have studied sequences within the *Alk* locus either by reporter activity assays [1, 17] or ChIP-on-chip analyses [16, 17]. Our analysis of reporter activity has identified regions upstream of *Alk* that are active in the AS, VM and epidermis. These coincide temporally with Alk protein expression, allowing us to define *Alk* VM, AS and epidermal enhancers located proximal to the *Alk-RB* promoter. High-throughput Y1H screens performed in this study identified a number of TFs that potentially bind to and regulate these regions of the *Alk* locus. In addition, a genome-wide ChIP-on-chip screen for mesodermal TFs occupancy identified a CRM upstream of the *Alk* locus that is active during mesoderm development [16]. This CRM maps to 2R:16,639,969..16,640,341 (relative to Dmel_Release_6 sequence assembly) and was described to be bound by mesodermal TFs including Bin, Bap, Mef2 and Tin and Twi [12, 15, 16, 29, 30]. However, none of these factors were found in our Y1H analysis. This may reflect additional requirements for binding of some TFs, which would preclude their identification by Y1H, such as heterodimerization with co-factors or post-translational modifications. Interestingly, homozygous mutants for *bin* and *bap* still express Alk protein in the VM, suggesting that while they may be involved in the modulation of *Alk* expression, additional factors are also important in the regulation of *Alk* expression in the VM.

One such factor could be the NK-4/msh-2 TF Tinman (Tin) which has been previously reported to bind CRMs at the *Alk* locus [16, 17]. Indeed, expression of *Alk* in the VM is affected in *tin* mutant embryos ([17], this study) however it is not clear if this occurs due to direct regulation of *Alk* expression by Tin or a general lack of induction of the VM lineage. Moreover, our analysis of *Alk*^*ΔOpaBS*^;*bap* double mutants uncovers a severe decrease in Alk protein in the VM suggesting only a minor direct contribution of Tin. Interestingly, *opa* has been reported to be directly regulated by Tin during heart development [17, 31] and Tin is critical for the expression of two key VM TFs *bin* and *bap* [4, 15]. Therefore it is likely that the importance of Tin for *Alk* expression relies on its activating potential for these *Alk*-regulating TFs. Interestingly, loss of *tin* does not affect *Alk* expression in the epidermis.

Reporter gene expression analyses suggest the VM *Alk* enhancer is located upstream of the *Alk-RB* isoform, in agreement with previously reported *AlkE301-lacZ* reporter spanning 1,984 bp (Fig 1A; S1A Fig) [17] and the 547 bp *MesoCRM-880* [16] that both cover the *AlkEB9* region. Our data suggests that *Alk-RB* expression can be activated through an upstream enhancer that is bound by Opa located within *AlkEB9*. We were also able to identify additional nearby enhancer elements in *AlkEB8* that integrate information from factors such as Bin and Bap that are critical to ensure precise and robust VM expression of *Alk*. Taken together with the earlier ChIP analyses from the Furlong and Frasch groups, our data suggest that Opa, along with mesodermal TFs such as Bin, Bap, Mef2 and Tin and Twi function in a combinatorial manner to drive robust expression of *Alk* in the VM (Fig 9I).

### Opa activates *Alk* transcription through the *AlkEB9* enhancer

Our efforts to identify novel TFs involved in *Alk* transcriptional control by *in vitro* Y1H assay resulted in a cluster of TFs potentially binding the *AlkEB9* sequence. Of those TF hits for which *UAS-transgenes* were available to test, only Opa was observed to induce cell autonomous expression of *Alk* when ectopically expressed. *opa* is a pair-rule gene [32] that encodes a zinc finger protein important during embryonic segmentation and midgut formation [26, 33, 34], as well as adult head morphogenesis by direct regulation of *decapentaplegic* (*dpp*) transcription [23, 35]. *opa* transcript is expressed in a spatially and temporally dynamic pattern, starting from stage 5 in a broad expression domain and from stage 11 onwards in two discrete domains in the VM corresponding to the first and third midgut constrictions [26, 33].

While Opa plays a role in the differentiating midgut musculature, with *opa* mutants exhibiting an interrupted VM unable to form midgut constrictions during embryogenesis [26], its role during segment formation presents a challenge when attempting to decipher the contribution of this TF more precisely. One component of this may be the regulation of *Alk* by Opa shown here. While we observed that *opa* mutants display lower levels of Alk protein in the VM, Jeb/Alk signaling is not abrogated, suggesting that while reduced, Alk protein levels are not reduced to levels under the threshold critical to drive Alk signaling. The lack of a critical role for Opa in the VM expression of *Alk* may reflect the importance of Alk signaling in this tissue for survival of the fly, where a more complex network of TFs may be employed to ensure rigorous *Alk* expression.

### A role for Bin and Bap in regulation of *Alk* transcription in the VM

Additional VM enhancer elements 5’ of *AlkEB9* in the *Alk* locus are regulated in part by Bin and Bap, two TFs that are critical for VM development. Thus multiple partially redundant enhancer regions are employed to safeguard VM expression of *Alk*, a phenomenon that has been observed in numerous genes expressed in the *Drosophila* embryonic muscle [36]. Moreover, while we have tested the role of Opa and the Opa binding sites in the *AlkEB9* region of the *Alk* locus in this work, we have done so under standard laboratory conditions, and as a result have not tested whether either Opa itself, *AlkEB9* or *AlkEB8* VM enhancers may play an increasingly critical role in *Alk* expression in more demanding environmental conditions, as it has been described for some *Drosophila* loci [37]. Although *Alk* is expressed in *bin* and *bap* mutants, our experiments combining deletion of the Opa binding region in *Alk* in a *bin* or *bap* mutant background suggest a combinatorial role for Bin, Bap and Opa driving VM expression of *Alk* [12–15]. Opa, Bin and Bap potentially act in combination with other TFs to control *Alk* transcription in the VM, as has been described for *sloppy paired-1* (*slp1*) activation in the somatic blastoderm in response to Opa and Runt [38]. In addition to direct regulation of *Alk* expression, Opa may also impact *Alk* expression via indirect mechanisms during embryogenesis.

Further complexity arises when the regulation of *opa* itself in the VM is considered. It is known that Dpp signaling restricts the VM spatial expression pattern of *opa* to PS6-8, with *dpp* mutants showing continuous *opa* expression throughout the VM [26]. Opa is also known to regulate *dpp* expression during adult head development [23]. In addition, *opa* is broadly expressed in the mesoderm at stage 6 potentially driving Dpp signaling. The Dpp mesodermal response consists of up-regulation of *tin* and *bap*, important regulatory genes in the dorsal mesoderm that essentially contribute to the specification of the VM [12, 39]. Similarly, Alk activity, the FoxF forkhead domain TF Bin and the Tbx1 Org-1, are also critical factors for expression of *dpp* in the VM and subsequent activation of Mad signaling in the midgut endoderm [40, 41]. Moreover, loss of *org-1*, whose expression is maintained by Alk signaling in the VM, results in decreased *opa* VM expression [4, 41], revealing a complex interplay of regulation where both Alk and Opa control each other’s expression in a spatially and temporally regulated manner.

Surprisingly, in addition to a non-essential role for Opa in the regulation of *Alk* transcription in the VM, in this work we have been able to identify a critical role for Opa in *Alk* expression in the AS and epidermis. Here, in contrast to the VM, Opa appears to be required and sufficient to drive *Alk* expression, although the functional significance of Alk in these tissues remains uncharacterized. Expression of the *AlkEB9-lacZ* reporter and derivatives in which the Opa binding sites have been mutated indicate that Opa has an important function in *Alk* transcription through the predicted *Opa BS*. This is supported by the absence of detectable Alk protein in the AS and epidermis of *Alk*^*ΔOpaBS*^ mutants, where the Opa binding sites within the *AlkEB9* enhancer have been deleted. Given that *Alk*^*ΔOpaBS*^ mutants are viable, it may be that Alk signaling is employed in a small population of non-essential cells that remain to be identified. Further work will be required to characterize the role of Alk in this context.

We have focused here on the regulation of *Alk* expression during embryonic development, however, Alk is also observed in larval and adult stages. Although Alk signaling does not seem to be critical for viability post-embryogenesis, a number of important roles in the nervous system have been described [42–47]. While we have not investigated the role of Opa, Bin or Bap in *Alk* expression at these other stages, nor in the CNS in this study, this would certainly be of interest to address in future experiments.

## Materials and methods

### *Drosophila* stocks and genetics

Standard *Drosophila* husbandry procedures were employed. *Drosophila* strains and crosses were maintained on a potato-meal based diet. Crosses were performed at controlled 60% humidity and 25°C conditions. Fly lines used in this study are: *UAS-Alk* [1], *UAS-GFP* (Bloomington 4775), *UAS-bap*.*ORF*.*3xHA* (FlyORF #F000006), *UAS-bin*.*ORF*.*3xHA* (FlyORF #F000281), *UAS-jeb* [6], *UAS-lacZ* (Bloomington 1776), *UAS-opa* [35], *UAS-opa*^*RNAi*^ (VDRC KK108975), *UAS-pnt*.*P1* (Bloomington 869), *UAS-side* (Bloomington 9679), *Alk*^*1*^ [2], *Alk*^*10*^ [2], *bap*^*208*^ [12], *Df(3R)ED6058* (Bloomington 24140), *bin*^*1*^ (Bloomington 1438), *Df(3L)BSC374* (Bloomington 24398), *opa*^*1*^ (Bloomington 3312 and 3222), *opa*^*8*^ (Bloomington 5335), *tin*^*346*^ [12], *AlkEI6*.*5-GAL4* [1], *bap3-GAL4* [15], *en2*.*4-GAL4* (Bloomington 30564), *prd-GAL4* (Bloomington 1947), *twi*.*2xPE-GAL4* (Bloomington 2517), *HandC-GFP* [48], *opa*^*3D246*^ [26], *Opa4opt-lacZ and Opa4opt-KO-lacZ* [23]. *Alk* alleles generated in this study are summarized in S1 Table.

Transgenic flies generated in this study: *AlkE4-GAL4*, *AlkE2*.*7-GAL4*, *AlkEB9-GAL4*, *eve*.*p*:*empty-lacZ*, *AlkEB6-lacZ*, *AlkEB7-lacZ*, *AlkEB8-lacZ*, *AlkEB9-lacZ*, *AlkEB10-lacZ*, *AlkEB11-lacZ*, *AlkEB9_OpaBS-lacZ*, *AlkEB9_OpaKO-lacZ*, *AlkEB8ΔBapBS-lacZ*, *AlkEB8∩EB9-lacZ*, *AlkEB8∩EB9+50flank-lacZ and AlkEB8∩EB9+100flank-lacZ*. Molecular details of the regions covered by these fragments are described in S2 Table. Genomic coordinates refer to the Dmel_Release_6 sequence assembly [49].

### Immunohistochemistry

Embryos were stained as described [1]. Primary antibodies used were: guinea pig anti-Alk (1:1000 [3]), rabbit anti-β-galactosidase (1:150; Cappel 0855976), chicken anti-β-galactosidase (1:200; Abcam ab9361), mouse anti-Fasciclin III (1:50; DSHB 7G10), rabbit anti-GFP (1:500; Abcam ab290), chicken anti-GFP (1:300; Abcam ab13970), mouse 16B12 anti-HA.11 (1:500; Covance #MMS-101P), rabbit anti-Org-1 (1:1000, this work), sheep anti-digoxygenin-AP fab fragment 1:4000 (Roche). Alexa Fluor^®^-conjugated secondary antibodies were from Jackson Immuno Research. Embryos were dehydrated in an ascending ethanol series before clearing and mounting in methylsalicylate.

Images were acquired with a Zeiss LSM800 confocal microscope or Axiocam 503 camera, processed and analyzed employing Zeiss ZEN2 (Blue Edition) imaging software. For analysis of protein levels, the laser, pinhole and PMT settings were adjusted on control siblings subsequently employed for imaging of mutant embryos.

Fluorescence intensity measurements were quantified using Zeiss ZEN2 (Blue Edition). In brief: mean fluorescence values were acquired from regions of interest (ROI), corresponding to the VM or epidermis (Alk staining) selected in confocal sections of stage 11 embryos. This mean fluorescent intensity was corrected using a background ROI chosen from a non-stained area. Measurements were taken from 10 embryos per sample analyzed. For statistical analysis we performed a one-way ANOVA using GraphPad Prism 6 software, where n.s. stands for non-significant, ***p≤0.001 and ****p≤0.0001. All plots are visualized as mean ±S.D.

### Generation of Org-1 antibodies

Recombinant N-terminal Org-1 protein was produced from *pET30a—Org-1-N* as generated by [50] was purified by His affinity chromatography and injected into rabbits for antibody generation (Genscript).

### *In situ* hybridization

For *in situ* hybridization, fragments of *Alk*, *gprs*, *CG5065* and *opa* were amplified from genomic DNA with the primer combinations shown in S3 Table. PCR products were cloned into the dual promoter PCRII TOPO vector (Invitrogen) and used as template to generate DIG-labeled *in situ* probes with SP6/T7 polymerases (Roche). *In situ* hybridization of antisense probes to embryos was carried out as previously described [51]. Samples were mounted in *in situ* mounting media (Electron Microscopy Sciences).

### High-throughput yeast one-hybrid screening

*pMW2*-vectors containing the different *Alk* putative CRMs were generated by regular cloning techniques (primer combinations shown in S4 Table) and integrated into the yeast genome as described [18]. Each DNA bait yeast strain was then transformed with a library of 647 *Drosophila* TFs fused to *GAL4*. Interaction was assessed by growing transformant yeast strains on selective plates followed by data analysis as previously described [18]. Briefly, selective growth of diploid yeast colonies was analyzed by the Matlab-based image-analysis program TIDY which quantifies bright spots, representing yeast colonies to the dark background. For every biological replicate in the screen, each bait-TF interaction was analyzed in four technical replicates resulting in quandrants of yeast colonies as shown for *AlkEB9* DNA bait in the results.

### Generation of transgenic flies

For generation of *lacZ* reporter flies, DNA sequences of for *AlkEB6* to *AlkEB11* were PCR amplified (S4 Table) and cloned into the *eve*.*p-lacZ*.*attB* vector [52]. In addition, the *AlkEB9* DNA bait was cloned into *pPT-GAL* vector (1225, DGRC) to generate the *AlkEB9-GAL4* construct. DNA sequences for *AlkEB8ΔBapBS-lacZ*, *AlkEB9_OpaBS-lacZ* and *AlkEB9_OpaKO-lacZ* were assembled by Genscript and cloned into *eve*.*p-lacZ*.*attB* vector for further *PhiC31* directed genome integration. For generation of *AlkE4-GAL4* and *AlkE2*.*7-GAL4* constructs, DNA genomic regions covering 2R:16,638,503..16,642,495 and 2R:16,638,510..16,640,834, respectively, were cloned into *pCaSpeR-DEST6* (1032, DGRC) by the Gateway system (primer combinations in S4 Table). Constructs were sequenced (GATC Biotech) and injected into *w*^*1118*^ flies, except for *attB* constructs which were injected into Bloomington 24482 and 24485, for *PhiC31* directed integration at 51C and 68E respectively (BestGene Inc.).

### Generation of *Alk* mutants

Deletions within the *Drosophila Alk* enhancer region were generated with CRISPR/Cas9 [53]. The *sgRNA* targeting sequences used (listed in S1 Table) were cloned into *pBFv-U6*.*2* expression vector (Genome Engineering Production Group at Harvard Medical School). Constructs expressing *sgRNA* were injected into *vasa (vas)-Cas9* (Bloomington 51323) embryos by BestGene Inc. Screening of deletion events was performed by PCR and further sequencing (GATC Biotech). For additional complementation tests we employed balanced *Alk*^*10*^ or *Df(2R)Exel7144* flies.

### Electrophoretic Mobility Shift Assay (EMSA)

DNA coding sequence of *opa* was synthesized (Genscript) in frame with carboxy-terminal OLLAS and 6xHis tags and cloned into the *pcDNA3*.*1(+)* mammalian expression vector. Binding of Opa to the *AlkEB9* was analyzed by a DNA binding assay on *dsDNA* oligonucleotides with cell lysates from HEK-293F cells expressing Opa-OLLAS. Binding reactions were performed as described in [54] containing 10 mM Tris-HCl (pH 8.0), 25 mM KCl and 1 mM DTT, 1 μg poly-dIdC (Sigma-Aldrich), 2.5% glycerol, 0.05% Triton X-100, 0.2 mM MgCl2 and the indicated 3’-end biotin labelled probe. After 20 min incubation at room temperature, reactions were separated on a 6% native TBE-PAGE in 0.5x TBE buffer at 100V. DNA was transferred to nylon+ membranes (Amersham), UV cross-linked to the membrane and detected by Chemoluminiscence Nucleic Acid Detection Module (Pierce) according to manufacturer’s indications. Competition assay was performed by addition of 100 fold molar excess of unlabeled competitor DNA to the reaction mix. Wild-type probes used for band shift experiments were *Opa_SELEX* and *Opa_JASPAR*. Mutated version were made according to for *Opa_SELEX* mutant [23], and in a similar manner for *Opa_JASPAR* mutant. All four EMSA probe sequences are shown in S3 Table.

### Chromatin immunoprecipitation

Chromatin was prepared from approximately 100 mg of pooled collections of fixed 3–4 hour embryos. The embryos were homogenized for 1 min in 10 mM EDTA and 50 mM Tris (pH 8.1). After addition of SDS to a final concentration of 1% and incubation on ice for 10 min, glass beads (150–200 μm) were added and the homogenates were sonicated to give sheared chromatin preparations with an average DNA size of 300–400 bp. Chromatin immuno-precipitation was performed largely as described previously [55] using an affinity-purified anti-Opa antibody raised against a truncated recombinant protein spanning from amino acids 125–507 of Opa, a region containing the DNA-binding zinc-fingers at a concentration of 0.5 μg/ml with 100 μg of chromatin in 1 ml of 0.01% SDS, 1% TritonX-100, 1 mM EDTA, 20 mM Tris, pH 8, 150 mM NaCl and 1x Protease Inhibitor Cocktail (Roche). After overnight incubation of the chromatin and antibody at 4°C, the mixture was incubated with Protein-A Agarose (Millipore) for 2 hours at room temperature, followed by low-salt, high salt and LiCl washes as used in the Chromatin Immunoprecipitation Assay Kit (Upstate Biotechnology). After heat reversal of protein-DNA crosslinks, protein digestion, phenol chloroform extraction and purification of the nucleic acids by ethanol precipitation the amount of recovered DNA was quantified using qPCR and a standard curve generated for each primer pair with a sample of nucleic acid purified from the input chromatin. The control primer pair produces a 115 bp amplicon located 12.4 kb upstream of odorant receptor 42b, a region devoid of modEncode hallmarks of *cis*-regulatory DNA sequences. The *DESE-Opa* primer pair produces a 209 bp amplicon from a central region of the *slp1 DESE* enhancer that requires Opa for expression [56]. The *Alk* primer pair produces a 140 bp amplicon that extends from 21 bp downstream of the *SELEX_OpaBS* to 53 bp upstream of the *JASPAR_OpaBS*. The ChIP values that are reported are percent precipitation relative to input DNA with error bars representing the mean ± S.D. from three technical replicates of the qPCR. The sequences of the primers are summarized in S3 Table and are as follows: *Or42b forward*: 5’ TCAAGCCGAACCCTCTAAAAT 3’, *Or42b reverse*: 5’ AACGCCAACAAACAGAAAATG 3’, DESE-Opa forward: 5’ TGCCGTTCGAGTCCTTTATT 3’, *DESE-Opa reverse*: 5’ CGGAGATCGGAAGGTTAGTG 3”, *Alk-OpaBS forward*: 5’ TTGTGCGTTTCACCAATCG 3’, Alk-OpaBS reverse: 5’ CGGACTAGCCACATCGAAC 3’.

## Supporting information

S1 Fig Schematic overview of the *Alk* locus summarizing this study. **(A)** Schematic representation of the *Alk* locus and its exon-intron structure, coding sequences shown in *white*. *GAL4* lines covering the 5’ region of the *Alk* locus are shown as *blue* lines. The *MesoCRM-880* and *AlkE301* CRMs identified in previous ChIP analyses are depicted as *grey* lines. DNA baits subjected to Y1H analysis—region shown in *red dashed lines*—are depicted as *black lines* (*AlkEB6 –AlkEB11*). A 2 kb close up window (*shaded yellow*) indicates the region shown in B and C. **(B)** Overview of *lacZ* reporters generated covering *AlkEB8* and *AlkEB9* (*light blue lines*). Predicted binding sites for mesoderm TFs (*pink*), Opa (*red*) and Bap (*grey*) are indicated. **(C)** Summary of the different deletions generated by CRISPR/Cas9 genome editing (*dashed lines*) and employed in this study.
(TIF)

S2 Fig *Alk* regulation in the presumptive Amnioserosa (AS). **(A)** *Alk* mRNA is observed in the dorsal most region of stage 6 *Drosophila* embryos, AS. **(B)** *Left panel*: *AlkEI6*.*5-GAL4* drives reporter expression in the AS (*red*), overlapping with Alk protein (*green*), in stage 10 embryos. *Right panel*: enlargement of boxed area, showing Alk protein (*green*) and *lacZ* expression (*red*) in AS cells. **(C)** *AlkEB8-lacZ* does not drive reporter expression in the AS (*lacZ* activity in *red*, Alk protein in *green*). **(D)** *AlkEB9-lacZ* drives reporter expression in the AS (*lacZ* activity in *red*, Alk protein in *green*). Scale bars: 50 μm.
(TIF)

S3 Fig Expression pattern of genes neighboring the *Alk* locus. **(A)** *gprs* transcripts are detected at later stages of embryogenesis in the developing CNS, with strong expression detected in the ventral midline. **(B)** *CG5065* transcripts were also only observed at later stages of embryogenesis in the foregut, hindgut and developing CNS. Scale bars: 50 μm.
(TIF)

S4 Fig Alk mRNA is absent in the VM and epidermis of the *Alk-RB* mutant, but is unaffected in the embryonic CNS. *Alk*^*ΔRB1*.*22*.*2*^ mutants lack mRNA in the VM at (B, stage 9) when compared to controls (A). *Alk* mRNA levels are unaffected in the CNS of *Alk*^*ΔRB1*.*22*.*2*^ mutant embryos (B, stage 16, compare with A). Scale bars: 50 μm.
(TIF)

S5 Fig Quantification of *AlkEB8-lacZ* and *AlkEB9-lacZ* in the VM. Both *AlkEB8-lacZ* and *AlkEB9-lacZ* are active in the VM, although *AlkEB8* displayed significantly less activity when compared to *AlkEB9*. Degrees of significance are denoted by ****p<0.0001 (n = 10 animals per genotype).
(TIF)

S6 Fig Ectopic expression of candidate TFs controlling *Alk* expression. **(A)** Stage 14 embryos display Alk protein in a segmented fashion in epidermis (*asterisks*), while *prd-GAL4* driver is active in every second cluster of Alk positive epidermal cells (*dashed box* indicates area of close-up). **(B)** Ectopic expression of *side* under *prd-GAL4* control does not lead to any changes in Alk protein in epidermis (*asterisks*; *dashed box* indicates area of close-up). **(C)** Overexpression of *pnt*.*P1* with *prd-GAL4* leads to a loss of Alk positive cells in epidermis (*asterisks*; *dashed box* indicates area of close-up). Scale bars: 50 μm and 10 μm (embryo and close ups, respectively).
(TIF)

S7 Fig Quantification of different reporter lines activity. *AlkEB9-lacZ*, *AlkEB9_OpaBS-lacZ* and *AlkEB9_OpaKO-lacZ* reporter activities were quantified in both the VM and the epidermis. *AlkEB9-lacZ* and *AlkEB9_OpaBS-lacZ* show a similar expression level in the epidermis, while *AlkEB9_OpaKO-lacZ* expression was significantly reduced. In the VM, expression of *AlkEB9-lacZ* was stronger than that of *AlkEB9_OpaBS-lacZ*, while mutation of the Opa binding sites in *AlkEB9_OpaKO-lacZ* resulted in a further reduction of activity. Degrees of significance are denoted by *n*.*s*. (not significant), *** (p≤0.001) and **** (p≤0.0001).
(TIF)

S8 Fig *opa mRNA* expression pattern and role in Alk signaling. **(A)** *opa* mRNA is expressed at high levels in a broad domain at stage 5. At later stages *opa* transcript is observed as 14 stripes of stronger expression alternating with stripes of weaker expression (stage 9). At stage 12, *opa* expression in discrete clusters of cells is observed in the VM (*arrowheads*), continuing at stage 14 where *opa* expression appears as two broad bands in the VM corresponding to parasegments 3–5 and 9–12 (*arrowheads*) and continuing to late embryogenesis. Embryos are oriented anterior left, dorsal up. **(B, C)** Org-1 (*red*) is observed in FCs nuclei in response to Jeb/Alk signaling (insets show area of close up). Levels are reduced, although still present, in *opa*^*1*^*/opa*^*8*^ mutants when compared with controls. Alk is shown in *green*. **(B’, C’)** Org-1 shown in *white*. **(D, E)** Similarly, *HandC-GFP* (*blue*) reflects Alk (*green*) signaling activity, but is not affected upon Opa overexpression in the VM with the *bap3-GAL4* driver (insets show area of close up). **(D’, E’)** Org-1 shown in *white*, **(D”, E”)** *HandC-GFP* shown in *white*. Stage 11 embryos are shown in B-E”. Scale bars: 50 μm.
(TIF)

S9 Fig Functional analysis of additional non-lethal CRISPR/Cas9 generated deletion mutants in the *Alk* locus. **(A, A’)** *Alk*^*ΔmesoBS*^ mutants do not show reduced levels of Alk protein in either the VM or epidermis (A, quantified in B). Furthermore, stage 16 homozygous mutant embryos show normal FC specification (inset, stage 11 embryo) and a chambered gut (A’, stage 16 embryo). **(B)** Quantification of Alk protein levels in *Alk*^*ΔmesoBS*^ mutants (*n*.*s*.–not significant; n = 10 animals per genotype). Scale bars: 50 μm.
(TIF)

S10 Fig Reporter activity analysis of the overlap region between *AlkEB8* and *AlkEB9*. **(A-C)** *lacZ* reporters corresponding to the overlapping region between *AlkEB8* and *AlkEB9* were analyzed for reporter activity (schematically depicted in S1 Fig). None of the three transgenic sequences containing increasing portions of the overlapping region between *AlkEB8* and *AlkEB9*, namely *EB8∩9-lacZ* (111 bp), *EB8∩9+50flank -lacZ* (211 bp) and *EB8∩9+100flank-lacZ* (311 bp) exhibit any detectable *lacZ* reporter activity during embryogenesis. Upper panels show *lacZ* reporter expression in *white*. Lower panels show a merged image of Alk protein shown in *green* and *lacZ* reporter expression in *red*. Scale bar: 50 μm.
(TIF)

S11 Fig *AlkEB9-lacZ* expression in *tin* mutant background. **(A-A’)** Expression of *AlkEB9-lacZ* in stage 11 *tin/ED6058* mutant embryos. Lateral view of epidermal Alk (*red*) and β-gal (*green*) (A). Deeper view reveals no detectable Alk (*red*) or lacZ(*green*) expression (A’). **(B-B’)** Stage 14 *tin/ED6058* mutant embryos do not exhibit VM structures (B’), however, both expression of *AlkEB9-lacZ* (*green*) and Alk protein (*red*) is visible in the epidermis (B). Scale bar: 50 μm.
(TIF)

S12 Fig Ectopic expression of either Bin or Bap does not drive ectopic *Alk* expression. **(A-C)** Ectopic expression of either *bap* (B) or *bin* (C) in the epidermis with *en-GAL4* does not result in a detectable increase in Alk protein levels. Alk protein shown in *red*, GFP in *green* (A), anti-HA in *green* (B, C). Scale bar: 50 μm.
(TIF)

S13 Fig Bin and Bap are required for full *AlkEB8-lacZ* activity. **(A-C’)** Expression of *AlkEB9-lacZ* in the VM is not altered in *bin*^*1*^*/BSC374* or *bap*^*208*^*/ED6058* embryos (*arrowheads*). **(D-F’)** In contrast, expression of *AlkEB8-lacZ* is mildly reduced in *bin*^*1*^*/BSC374* animals and undetectable in *bap*^*208*^*/ED6058* embryos (*arrowheads*). Alk protein is shown in *green*, *lacZ* reporter expression in *red*, FasIII shown in *blue*. Scale bar: 50 μm.
(TIF)

S1 Table Summary of CRISPR/Cas9 mutants generated in this study. Molecular details of each mutant and characterization of their Alk expression patterns are included.
(XLSX)

S2 Table Summary of transgenic *Drosophila* generated in this study. Molecular details are included.
(XLSX)

S3 Table Oligonucleotide sequences employed for *in situ*, EMSA and ChIP analysis. (XLSX)

S4 Table Primer combinations used to clone DNA-baits into either *pMW2* for yeast one-hybrid screening or *eve*.*p-lacZ*.*attB* for reporter analysis. (XLSX)

## References

[1] Loren CE, Scully A, Grabbe C, Edeen PT, Thomas J, McKeown M, et al Identification and characterization of DAlk: a novel Drosophila melanogaster RTK which drives ERK activation in vivo. Genes Cells. 2001;6(6):531–44. 11442633 10.1046/j.1365-2443.2001.00440.x PMC1975818

[2] Loren CE, Englund C, Grabbe C, Hallberg B, Hunter T, Palmer RH. A crucial role for the Anaplastic lymphoma kinase receptor tyrosine kinase in gut development in Drosophila melanogaster. EMBO Rep. 2003;4(8):781–6. Epub 2003/07/12. 10.1038/sj.embor.embor897 12855999 PMC1326337

[3] Englund C, Loren CE, Grabbe C, Varshney GK, Deleuil F, Hallberg B, et al Jeb signals through the Alk receptor tyrosine kinase to drive visceral muscle fusion. Nature. 2003;425(6957):512–6. 10.1038/nature01950 14523447

[4] Lee HH, Norris A, Weiss JB, Frasch M. Jelly belly protein activates the receptor tyrosine kinase Alk to specify visceral muscle pioneers. Nature. 2003;425(6957):507–12. 10.1038/nature01916 14523446

[5] Stute C, Schimmelpfeng K, Renkawitz-Pohl R, Palmer RH, Holz A. Myoblast determination in the somatic and visceral mesoderm depends on Notch signalling as well as on milliways(mili(Alk)) as receptor for Jeb signalling. Development. 2004;131(4):743–54. 10.1242/dev.00972 14757637

[6] Varshney GK, Palmer RH. The bHLH transcription factor Hand is regulated by Alk in the Drosophila embryonic gut. Biochem Biophys Res Commun. 2006;351(4):839–46. 10.1016/j.bbrc.2006.10.117 17094947

[7] Bour BA, Chakravarti M, West JM, Abmayr SM. Drosophila SNS, a member of the immunoglobulin superfamily that is essential for myoblast fusion. Genes Dev. 2000;14(12):1498–511. 10859168 PMC316690

[8] Eriksson T, Varshney G, Aspenstrom P, Palmer RH. Characterisation of the role of Vrp1 in cell fusion during the development of visceral muscle of Drosophila melanogaster. BMC Dev Biol. 2010;10:86 Epub 2010/08/13. 10.1186/1471-213X-10-86 20701765 PMC2931478

[9] Massarwa R, Carmon S, Shilo BZ, Schejter ED. WIP/WASp-based actin-polymerization machinery is essential for myoblast fusion in Drosophila. Dev Cell. 2007;12(4):557–69. Epub 2007/04/11. 10.1016/j.devcel.2007.01.016 17419994

[10] Kim S, Shilagardi K, Zhang S, Hong SN, Sens KL, Bo J, et al A critical function for the actin cytoskeleton in targeted exocytosis of prefusion vesicles during myoblast fusion. Dev Cell. 2007;12(4):571–86. Epub 2007/04/11. 10.1016/j.devcel.2007.02.019 17419995

[11] Popichenko D, Hugosson F, Sjogren C, Dogru M, Yamazaki Y, Wolfstetter G, et al Jeb/Alk signalling regulates the Lame duck GLI family transcription factor in the Drosophila visceral mesoderm. Development. 2013;140(15):3156–66. 10.1242/dev.094466 23824577

[12] Azpiazu N, Frasch M. tinman and bagpipe: two homeo box genes that determine cell fates in the dorsal mesoderm of Drosophila. Genes Dev. 1993;7(7B):1325–40. 8101173 10.1101/gad.7.7b.1325

[13] Bodmer R. The gene tinman is required for specification of the heart and visceral muscles in Drosophila. Development. 1993;118(3):719–29. 7915669 10.1242/dev.118.3.719

[14] Riechmann V, Irion U, Wilson R, Grosskortenhaus R, Leptin M. Control of cell fates and segmentation in the Drosophila mesoderm. Development. 1997;124(15):2915–22. 9247334 10.1242/dev.124.15.2915

[15] Zaffran S, Kuchler A, Lee HH, Frasch M. biniou (FoxF), a central component in a regulatory network controlling visceral mesoderm development and midgut morphogenesis in Drosophila. Genes Dev. 2001;15(21):2900–15. Epub 2001/11/03. 11691840 10.1101/gad.917101 PMC312807

[16] Zinzen RP, Girardot C, Gagneur J, Braun M, Furlong EE. Combinatorial binding predicts spatio-temporal cis-regulatory activity. Nature. 2009;462(7269):65–70. 10.1038/nature08531 19890324

[17] Jin H, Stojnic R, Adryan B, Ozdemir A, Stathopoulos A, Frasch M. Genome-wide screens for in vivo Tinman binding sites identify cardiac enhancers with diverse functional architectures. PLoS Genet. 2013;9(1):e1003195 10.1371/journal.pgen.1003195 23326246 PMC3542182

[18] Hens K, Feuz JD, Isakova A, Iagovitina A, Massouras A, Bryois J, et al Automated protein-DNA interaction screening of Drosophila regulatory elements. Nature methods. 2011;8(12):1065–70. 10.1038/nmeth.1763 22037703 PMC3929264

[19] Jinek M, Chylinski K, Fonfara I, Hauer M, Doudna JA, Charpentier E. A programmable dual-RNA-guided DNA endonuclease in adaptive bacterial immunity. Science. 2012;337(6096):816–21. 10.1126/science.1225829 22745249 PMC6286148

[20] Gratz SJ, Cummings AM, Nguyen JN, Hamm DC, Donohue LK, Harrison MM, et al Genome engineering of Drosophila with the CRISPR RNA-guided Cas9 nuclease. Genetics. 2013;194(4):1029–35. 10.1534/genetics.113.152710 23709638 PMC3730909

[21] Kondo S, Ueda R. Highly improved gene targeting by germline-specific Cas9 expression in Drosophila. Genetics. 2013;195(3):715–21. 10.1534/genetics.113.156737 24002648 PMC3813859

[22] Mathelier A, Zhao X, Zhang AW, Parcy F, Worsley-Hunt R, Arenillas DJ, et al JASPAR 2014: an extensively expanded and updated open-access database of transcription factor binding profiles. Nucleic acids research. 2014;42(Database issue):D142–7. 10.1093/nar/gkt997 24194598 PMC3965086

[23] Sen A, Stultz BG, Lee H, Hursh DA. Odd paired transcriptional activation of decapentaplegic in the Drosophila eye/antennal disc is cell autonomous but indirect. Dev Biol. 2010;343(1–2):167–77. 10.1016/j.ydbio.2010.04.003 20403347

[24] Siepel A, Haussler D. Combining phylogenetic and hidden Markov models in biosequence analysis. J Comput Biol. 2004;11(2–3):413–28. 10.1089/1066527041410472 15285899

[25] Siepel A, Bejerano G, Pedersen JS, Hinrichs AS, Hou M, Rosenbloom K, et al Evolutionarily conserved elements in vertebrate, insect, worm, and yeast genomes. Genome Res. 2005;15(8):1034–50. 10.1101/gr.3715005 16024819 PMC1182216

[26] Cimbora DM, Sakonju S. Drosophila midgut morphogenesis requires the function of the segmentation gene odd-paired. Dev Biol. 1995;169(2):580–95. 10.1006/dbio.1995.1171 7781900

[27] Kpebe A, Rabinow L. Alternative promoter usage generates multiple evolutionarily conserved isoforms of Drosophila DOA kinase. Genesis. 2008;46(3):132–43. 10.1002/dvg.20374 18327787

[28] Kim SY, Renihan MK, Boulianne GL. Characterization of big bang, a novel gene encoding for PDZ domain-containing proteins that are dynamically expressed throughout Drosophila development. Gene Expr Patterns. 2006;6(5):504–18. 10.1016/j.modgep.2005.10.009 16423565

[29] Leptin M. twist and snail as positive and negative regulators during Drosophila mesoderm development. Genes Dev. 1991;5(9):1568–76. 1884999 10.1101/gad.5.9.1568

[30] Nguyen HT, Bodmer R, Abmayr SM, McDermott JC, Spoerel NA. D-mef2: a Drosophila mesoderm-specific MADS box-containing gene with a biphasic expression profile during embryogenesis. Proceedings of the National Academy of Sciences of the United States of America. 1994;91(16):7520–4. 8052612 10.1073/pnas.91.16.7520 PMC44433

[31] Liu YH, Jakobsen JS, Valentin G, Amarantos I, Gilmour DT, Furlong EE. A systematic analysis of Tinman function reveals Eya and JAK-STAT signaling as essential regulators of muscle development. Dev Cell. 2009;16(2):280–91. 10.1016/j.devcel.2009.01.006 19217429

[32] Jurgens G, Wieschaus E, Nussleinvolhard C, Kluding H. Mutations Affecting the Pattern of the Larval Cuticle in Drosophila-Melanogaster 2. Zygotic Loci on the 3rd Chromosome. Roux Arch Dev Biol. 1984;193(5):283–95. 10.1007/BF00848157 28305338

[33] Benedyk MJ, Mullen JR, DiNardo S. odd-paired: a zinc finger pair-rule protein required for the timely activation of engrailed and wingless in Drosophila embryos. Genes & development. 1994;8(1):105–17. 8288124 10.1101/gad.8.1.105

[34] Azpiazu N, Lawrence PA, Vincent JP, Frasch M. Segmentation and specification of the Drosophila mesoderm. Genes Dev. 1996;10(24):3183–94. 8985186 10.1101/gad.10.24.3183

[35] Lee H, Stultz BG, Hursh DA. The Zic family member, odd-paired, regulates the Drosophila BMP, decapentaplegic, during adult head development. Development. 2007;134(7):1301–10. 10.1242/dev.02807 17329368

[36] Cannavo E, Khoueiry P, Garfield DA, Geeleher P, Zichner T, Gustafson EH, et al Shadow Enhancers Are Pervasive Features of Developmental Regulatory Networks. Current biology: CB. 2016;26(1):38–51. 10.1016/j.cub.2015.11.034 26687625 PMC4712172

[37] Frankel N, Davis GK, Vargas D, Wang S, Payre F, Stern DL. Phenotypic robustness conferred by apparently redundant transcriptional enhancers. Nature. 2010;466(7305):490–3. 10.1038/nature09158 20512118 PMC2909378

[38] Swantek D, Gergen JP. Ftz modulates Runt-dependent activation and repression of segment-polarity gene transcription. Development. 2004;131(10):2281–90. 10.1242/dev.01109 15102703

[39] Lee HH, Frasch M. Nuclear integration of positive Dpp signals, antagonistic Wg inputs and mesodermal competence factors during Drosophila visceral mesoderm induction. Development. 2005;132(6):1429–42. 10.1242/dev.01687 15750188

[40] Shirinian M, Varshney G, Loren CE, Grabbe C, Palmer RH. Drosophila Anaplastic Lymphoma Kinase regulates Dpp signalling in the developing embryonic gut. Differentiation. 2007;75(5):418–26. Epub 2007/02/09. 10.1111/j.1432-0436.2006.00148.x 17286600

[41] Schaub C, Frasch M. Org-1 is required for the diversification of circular visceral muscle founder cells and normal midgut morphogenesis. Developmental biology. 2013;376(2):245–59. 10.1016/j.ydbio.2013.01.022 23380635 PMC3602240

[42] Bazigou E, Apitz H, Johansson J, Loren CE, Hirst EM, Chen PL, et al Anterograde Jelly belly and Alk receptor tyrosine kinase signaling mediates retinal axon targeting in Drosophila. Cell. 2007;128(5):961–75. 10.1016/j.cell.2007.02.024 17350579

[43] Cheng LY, Bailey AP, Leevers SJ, Ragan TJ, Driscoll PC, Gould AP. Anaplastic lymphoma kinase spares organ growth during nutrient restriction in Drosophila. Cell. 2011;146(3):435–47. Epub 2011/08/06. 10.1016/j.cell.2011.06.040 21816278

[44] Gouzi JY, Moressis A, Walker JA, Apostolopoulou AA, Palmer RH, Bernards A, et al The receptor tyrosine kinase Alk controls neurofibromin functions in Drosophila growth and learning. PLoS Genet. 2011;7(9):e1002281 Epub 2011/09/29. 10.1371/journal.pgen.1002281 21949657 PMC3174217

[45] Lasek AW, Lim J, Kliethermes CL, Berger KH, Joslyn G, Brush G, et al An evolutionary conserved role for anaplastic lymphoma kinase in behavioral responses to ethanol. PLoS One. 2011;6(7):e22636 Epub 2011/07/30. 10.1371/journal.pone.0022636 21799923 PMC3142173

[46] Okamoto N, Nishimura T. Signaling from Glia and Cholinergic Neurons Controls Nutrient-Dependent Production of an Insulin-like Peptide for Drosophila Body Growth. Dev Cell. 2015;35(3):295–310. 10.1016/j.devcel.2015.10.003 26555050

[47] Rohrbough J, Broadie K. Anterograde Jelly belly ligand to Alk receptor signaling at developing synapses is regulated by Mind the gap. Development. 2010;137(20):3523–33. 10.1242/dev.047878 20876658 PMC2947762

[48] Sellin J, Albrecht S, Kolsch V, Paululat A. Dynamics of heart differentiation, visualized utilizing heart enhancer elements of the Drosophila melanogaster bHLH transcription factor Hand. Gene Expression Patterns. 2006;6(4):360–75. 10.1016/j.modgep.2005.09.012 16455308

[49] Hoskins RA, Carlson JW, Wan KH, Park S, Mendez I, Galle SE, et al The Release 6 reference sequence of the Drosophila melanogaster genome. Genome Res. 2015;25(3):445–58. 10.1101/gr.185579.114 25589440 PMC4352887

[50] Schaub C, Nagaso H, Jin H, Frasch M. Org-1, the Drosophila ortholog of Tbx1, is a direct activator of known identity genes during muscle specification. Development. 2012;139(5):1001–12. Epub 2012/02/10. 10.1242/dev.073890 22318630 PMC3274361

[51] Pfeifer K, Dorresteijn AW, Frobius AC. Activation of Hox genes during caudal regeneration of the polychaete annelid Platynereis dumerilii. Development genes and evolution. 2012;222(3):165–79. 10.1007/s00427-012-0402-z 22569931

[52] Liberman LM, Stathopoulos A. Design flexibility in cis-regulatory control of gene expression: synthetic and comparative evidence. Developmental biology. 2009;327(2):578–89. 10.1016/j.ydbio.2008.12.020 19135437 PMC2746413

[53] Ren X, Sun J, Housden BE, Hu Y, Roesel C, Lin S, et al Optimized gene editing technology for Drosophila melanogaster using germ line-specific Cas9. Proceedings of the National Academy of Sciences of the United States of America. 2013;110(47):19012–7. 10.1073/pnas.1318481110 24191015 PMC3839733

[54] Kremnev D, Strand A. Plastid encoded RNA polymerase activity and expression of photosynthesis genes required for embryo and seed development in Arabidopsis. Frontiers in plant science. 2014;5:385 10.3389/fpls.2014.00385 25161659 PMC4130184

[55] Wang X, Lee C, Gilmour DS, Gergen JP. Transcription elongation controls cell fate specification in the Drosophila embryo. Genes Dev. 2007;21(9):1031–6. 10.1101/gad.1521207 17473169 PMC1855229

[56] Prazak L, Fujioka M, Gergen JP. Non-additive interactions involving two distinct elements mediate sloppy-paired regulation by pair-rule transcription factors. Dev Biol. 2010;344(2):1048–59. 10.1016/j.ydbio.2010.04.026 20435028 PMC2914134

