# Supplementary figures and images for: The Zic family homologue Odd-paired regulates Alk expression in Drosophila
Source: PLoS Genet. 2017 Apr 3;13(4):e1006617. doi: 10.1371/journal.pgen.1006617 (PMC5393633; doi:10.1371/journal.pgen.1006617)

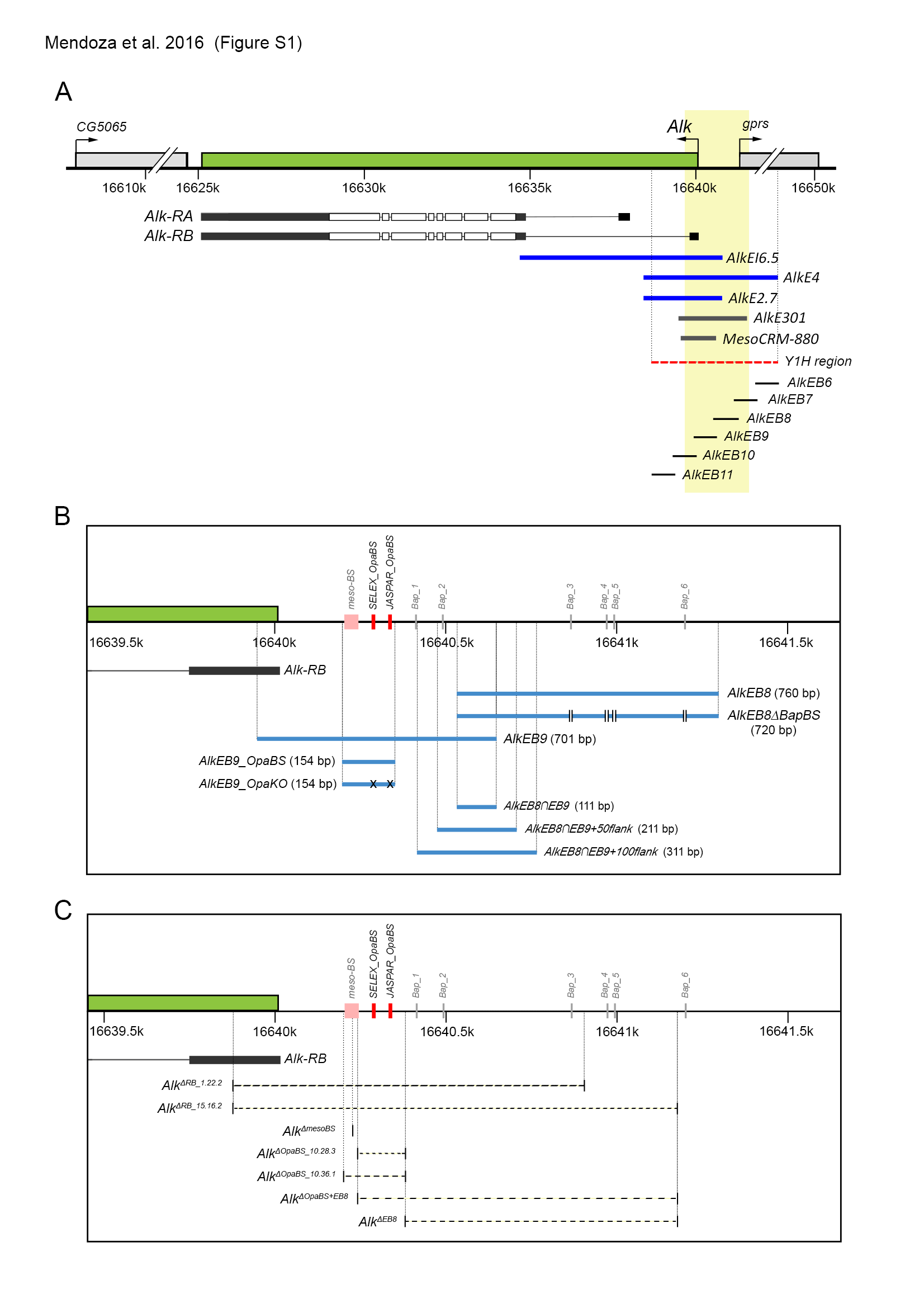

Supplement: S1 Fig — (A) Schematic representation of the Alk locus and its exon-intron structure, coding sequences shown in white. GAL4 lines covering the 5’ region of the Alk locus are shown as blue lines. The MesoCRM-880 and AlkE301 CRMs identified in previous ChIP analyses are depicted as grey lines. DNA baits subjected to Y1H analysis—region shown in red dashed lines—are depicted as black lines (AlkEB6 –AlkEB11). A 2 kb close up window (shaded yellow) indicates the region shown in B and C. (B) Overview of lacZ reporters generated covering AlkEB8 and AlkEB9 (light blue lines). Predicted binding sites for mesoderm TFs (pink), Opa (red) and Bap (grey) are indicated. (C) Summary of the different deletions generated by CRISPR/Cas9 genome editing (dashed lines) and employed in this study. (TIF) [file pgen.1006617.s001.tif]

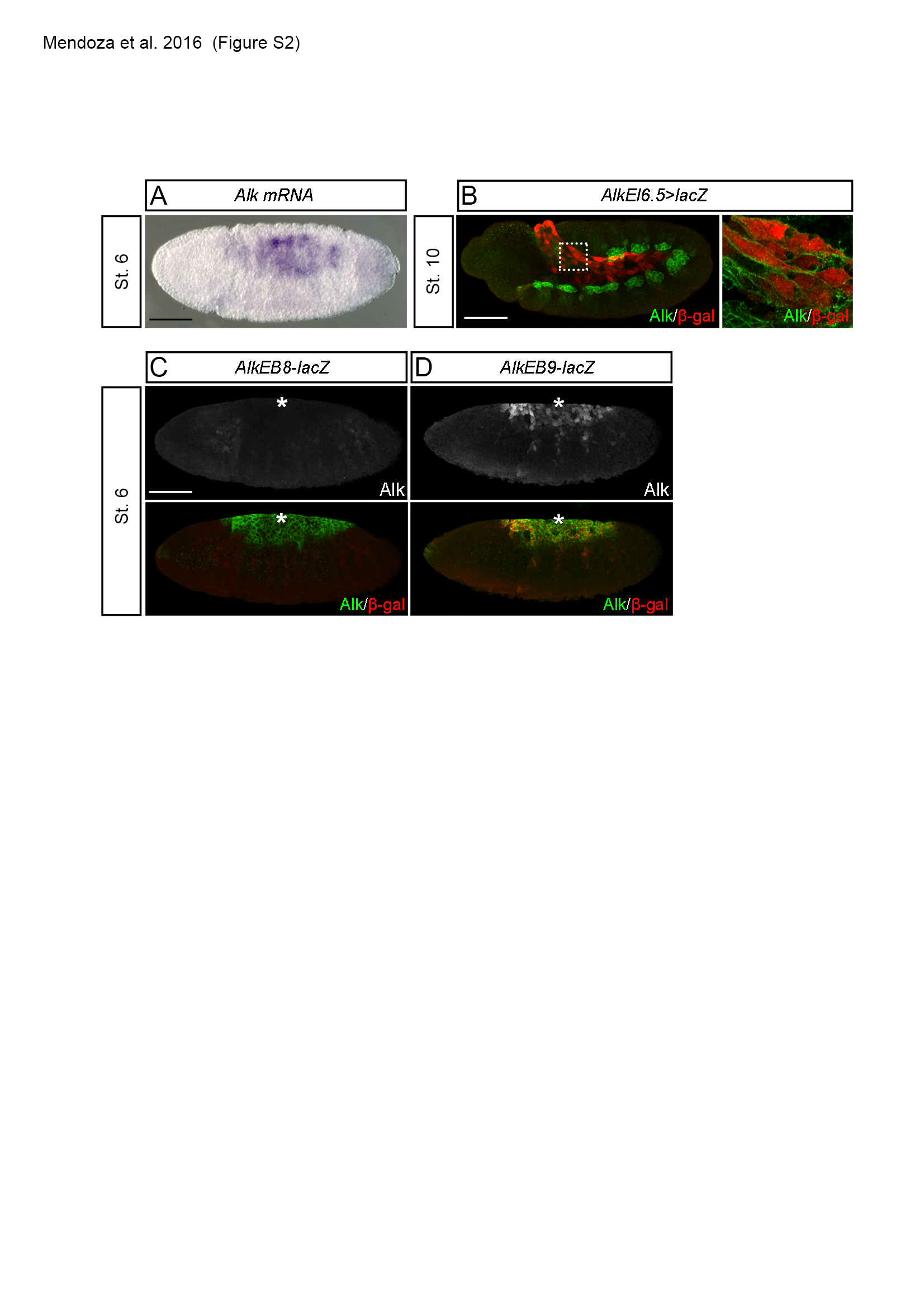

Supplement: S2 Fig — (A) Alk mRNA is observed in the dorsal most region of stage 6 Drosophila embryos, AS. (B) Left panel: AlkEI6.5-GAL4 drives reporter expression in the AS (red), overlapping with Alk protein (green), in stage 10 embryos. Right panel: enlargement of boxed area, showing Alk protein (green) and lacZ expression (red) in AS cells. (C) AlkEB8-lacZ does not drive reporter expression in the AS (lacZ activity in red, Alk protein in green). (D) AlkEB9-lacZ drives reporter expression in the AS (lacZ activity in red, Alk protein in green). Scale bars: 50 μm. (TIF) [file pgen.1006617.s002.tif]

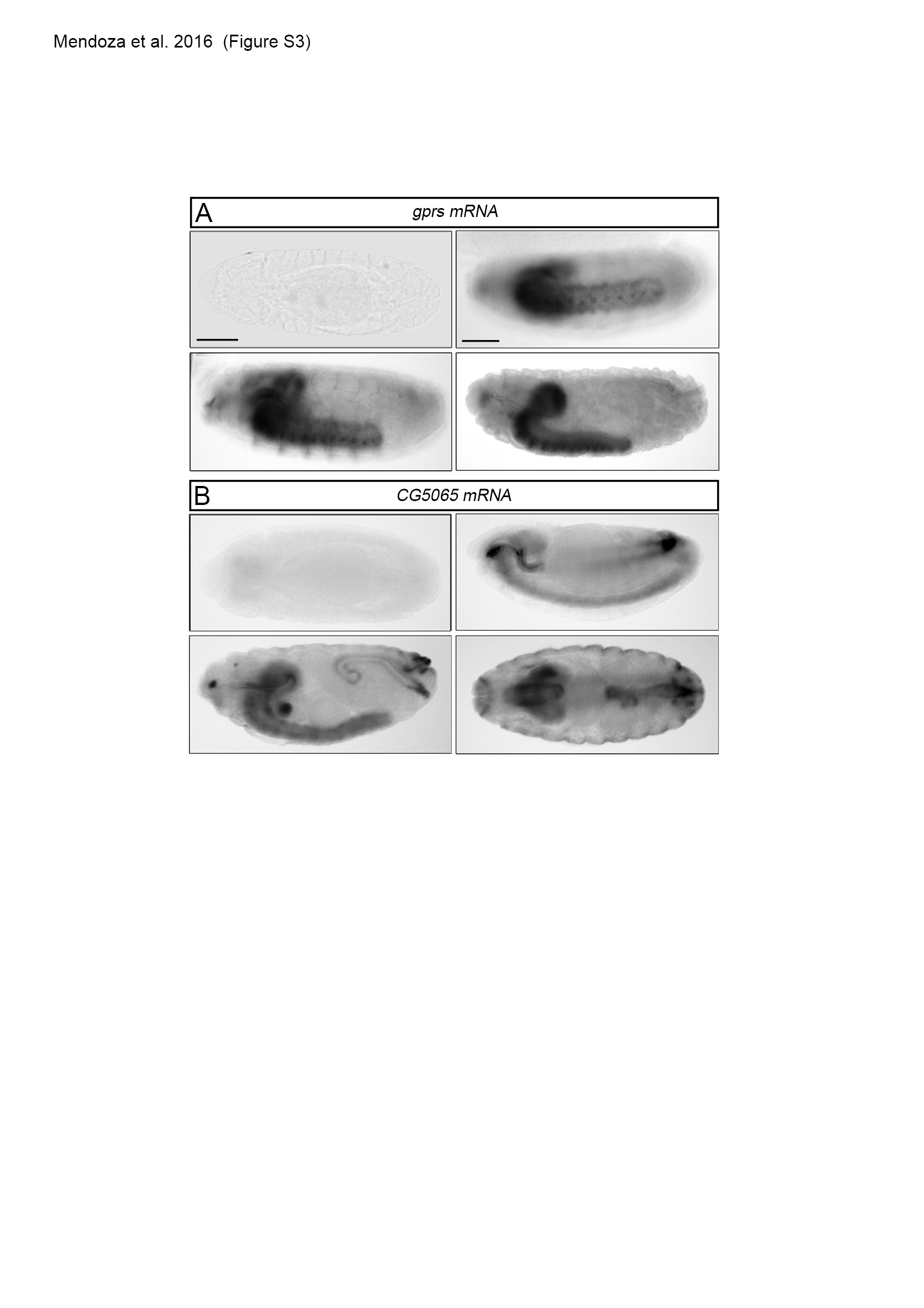

Supplement: S3 Fig — (A) gprs transcripts are detected at later stages of embryogenesis in the developing CNS, with strong expression detected in the ventral midline. (B) CG5065 transcripts were also only observed at later stages of embryogenesis in the foregut, hindgut and developing CNS. Scale bars: 50 μm. (TIF) [file pgen.1006617.s003.tif]

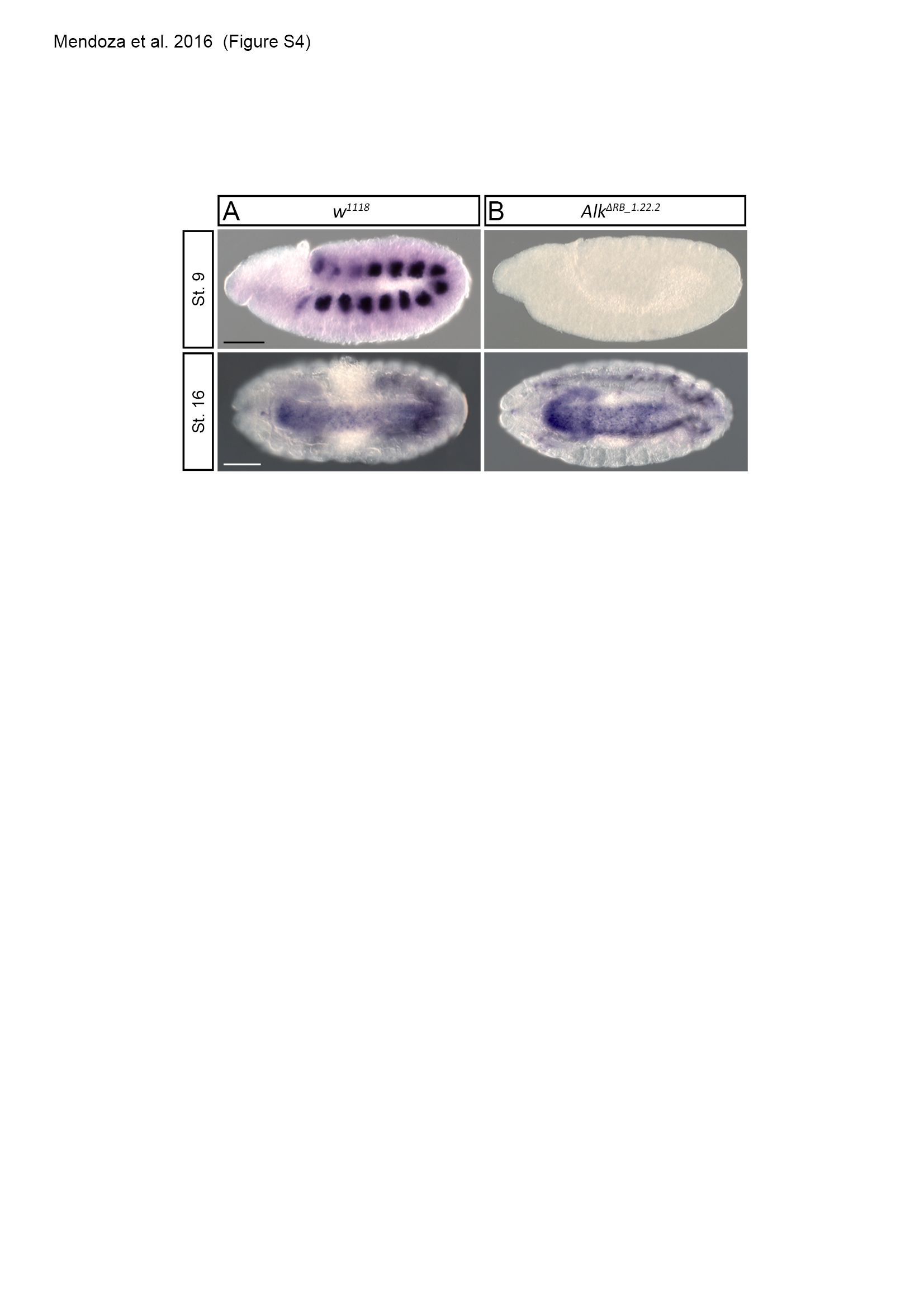

Supplement: S4 Fig — AlkΔRB1.22.2 mutants lack mRNA in the VM at (B, stage 9) when compared to controls (A). Alk mRNA levels are unaffected in the CNS of AlkΔRB1.22.2 mutant embryos (B, stage 16, compare with A). Scale bars: 50 μm. (TIF) [file pgen.1006617.s004.tif]

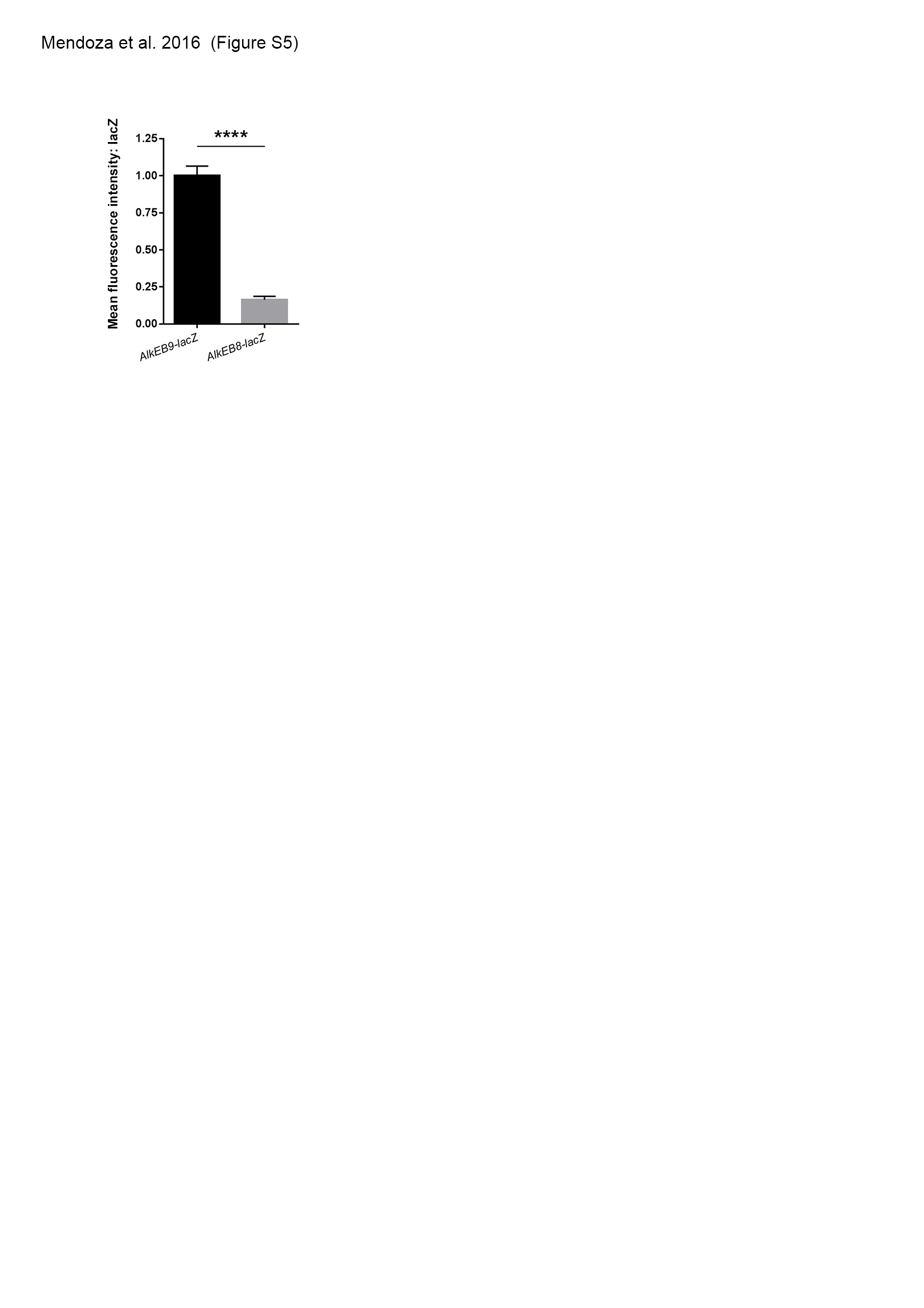

Supplement: S5 Fig — Both AlkEB8-lacZ and AlkEB9-lacZ are active in the VM, although AlkEB8 displayed significantly less activity when compared to AlkEB9. Degrees of significance are denoted by ****p<0.0001 (n = 10 animals per genotype). (TIF) [file pgen.1006617.s005.tif]

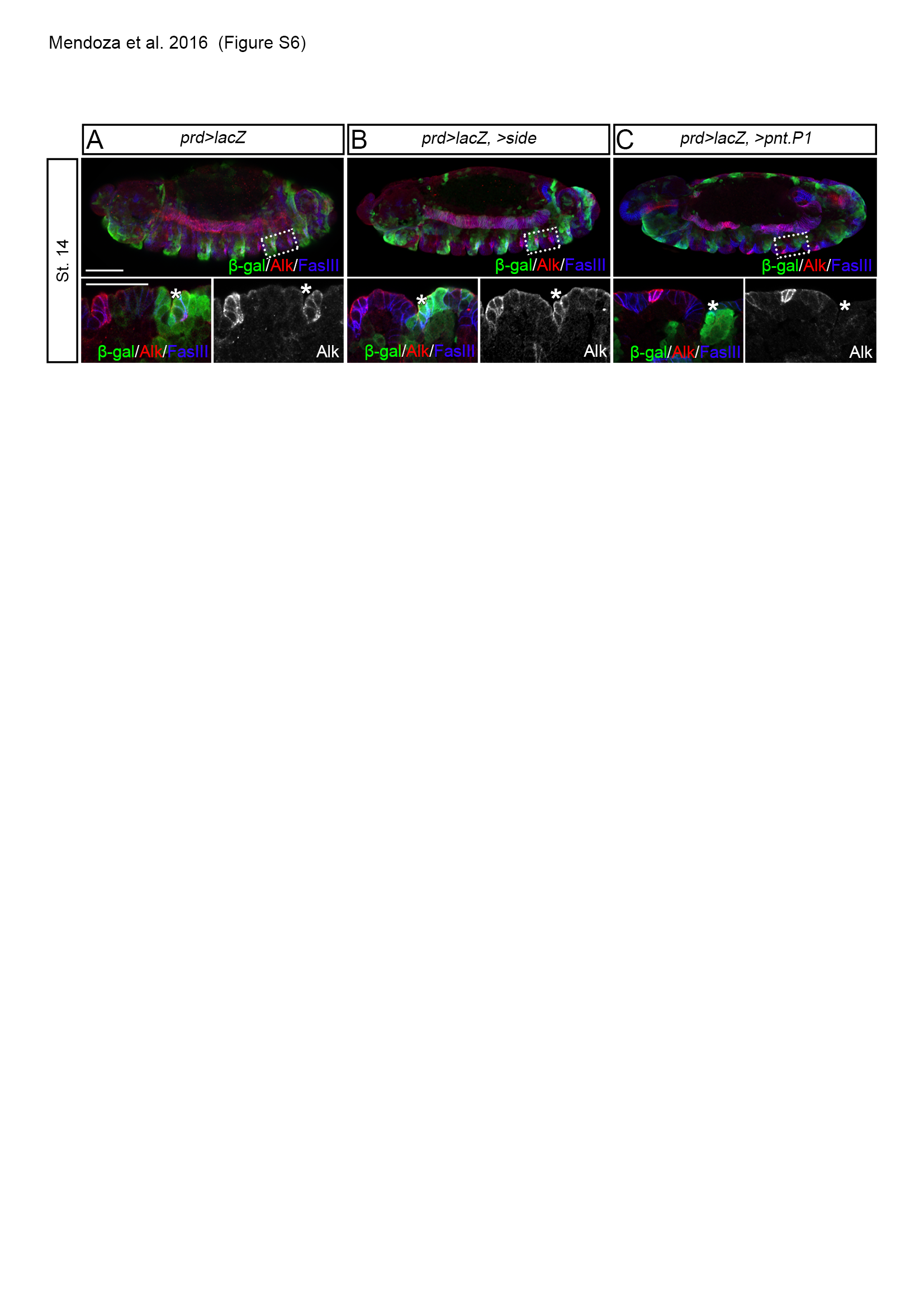

Supplement: S6 Fig — (A) Stage 14 embryos display Alk protein in a segmented fashion in epidermis (asterisks), while prd-GAL4 driver is active in every second cluster of Alk positive epidermal cells (dashed box indicates area of close-up). (B) Ectopic expression of side under prd-GAL4 control does not lead to any changes in Alk protein in epidermis (asterisks; dashed box indicates area of close-up). (C) Overexpression of pnt.P1 with prd-GAL4 leads to a loss of Alk positive cells in epidermis (asterisks; dashed box indicates area of close-up). Scale bars: 50 μm and 10 μm (embryo and close ups, respectively). (TIF) [file pgen.1006617.s006.tif]

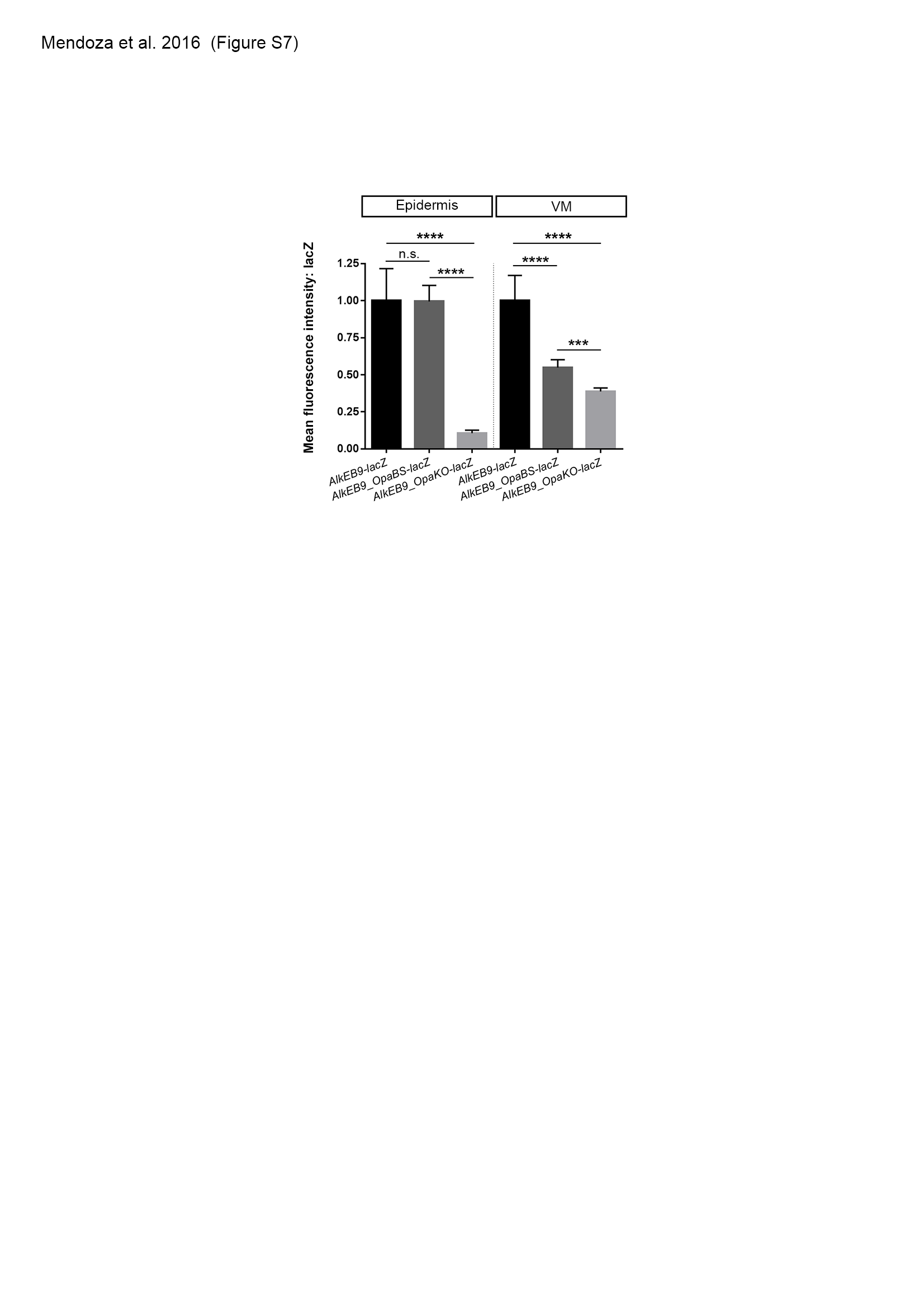

Supplement: S7 Fig — AlkEB9-lacZ, AlkEB9_OpaBS-lacZ and AlkEB9_OpaKO-lacZ reporter activities were quantified in both the VM and the epidermis. AlkEB9-lacZ and AlkEB9_OpaBS-lacZ show a similar expression level in the epidermis, while AlkEB9_OpaKO-lacZ expression was significantly reduced. In the VM, expression of AlkEB9-lacZ was stronger than that of AlkEB9_OpaBS-lacZ, while mutation of the Opa binding sites in AlkEB9_OpaKO-lacZ resulted in a further reduction of activity. Degrees of significance are denoted by n.s. (not significant), *** (p≤0.001) and **** (p≤0.0001). (TIF) [file pgen.1006617.s007.tif]

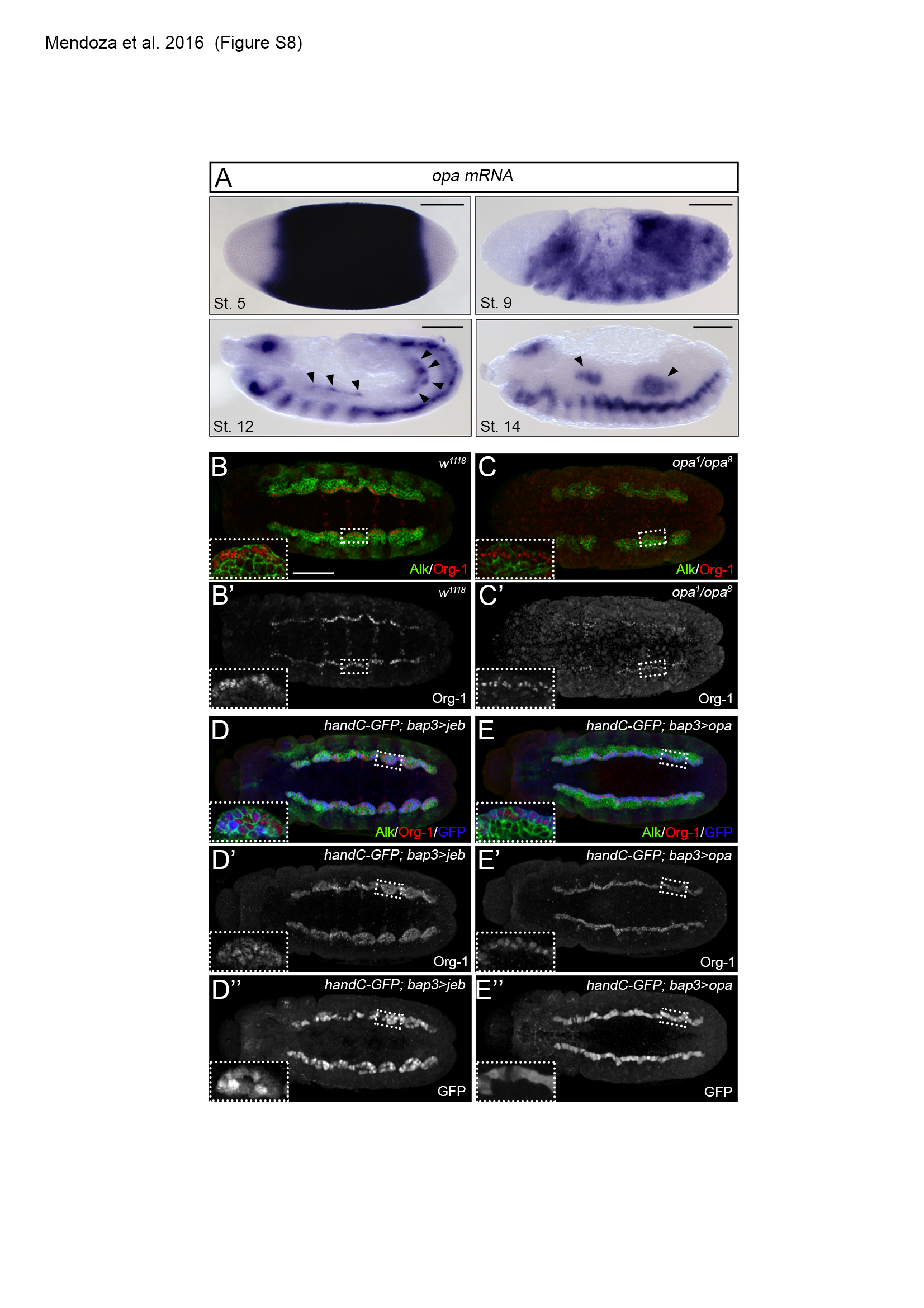

Supplement: S8 Fig — (A) opa mRNA is expressed at high levels in a broad domain at stage 5. At later stages opa transcript is observed as 14 stripes of stronger expression alternating with stripes of weaker expression (stage 9). At stage 12, opa expression in discrete clusters of cells is observed in the VM (arrowheads), continuing at stage 14 where opa expression appears as two broad bands in the VM corresponding to parasegments 3–5 and 9–12 (arrowheads) and continuing to late embryogenesis. Embryos are oriented anterior left, dorsal up. (B, C) Org-1 (red) is observed in FCs nuclei in response to Jeb/Alk signaling (insets show area of close up). Levels are reduced, although still present, in opa1/opa8 mutants when compared with controls. Alk is shown in green. (B’, C’) Org-1 shown in white. (D, E) Similarly, HandC-GFP (blue) reflects Alk (green) signaling activity, but is not affected upon Opa overexpression in the VM with the bap3-GAL4 driver (insets show area of close up). (D’, E’) Org-1 shown in white, (D”, E”) HandC-GFP shown in white. Stage 11 embryos are shown in B-E”. Scale bars: 50 μm. (TIF) [file pgen.1006617.s008.tif]

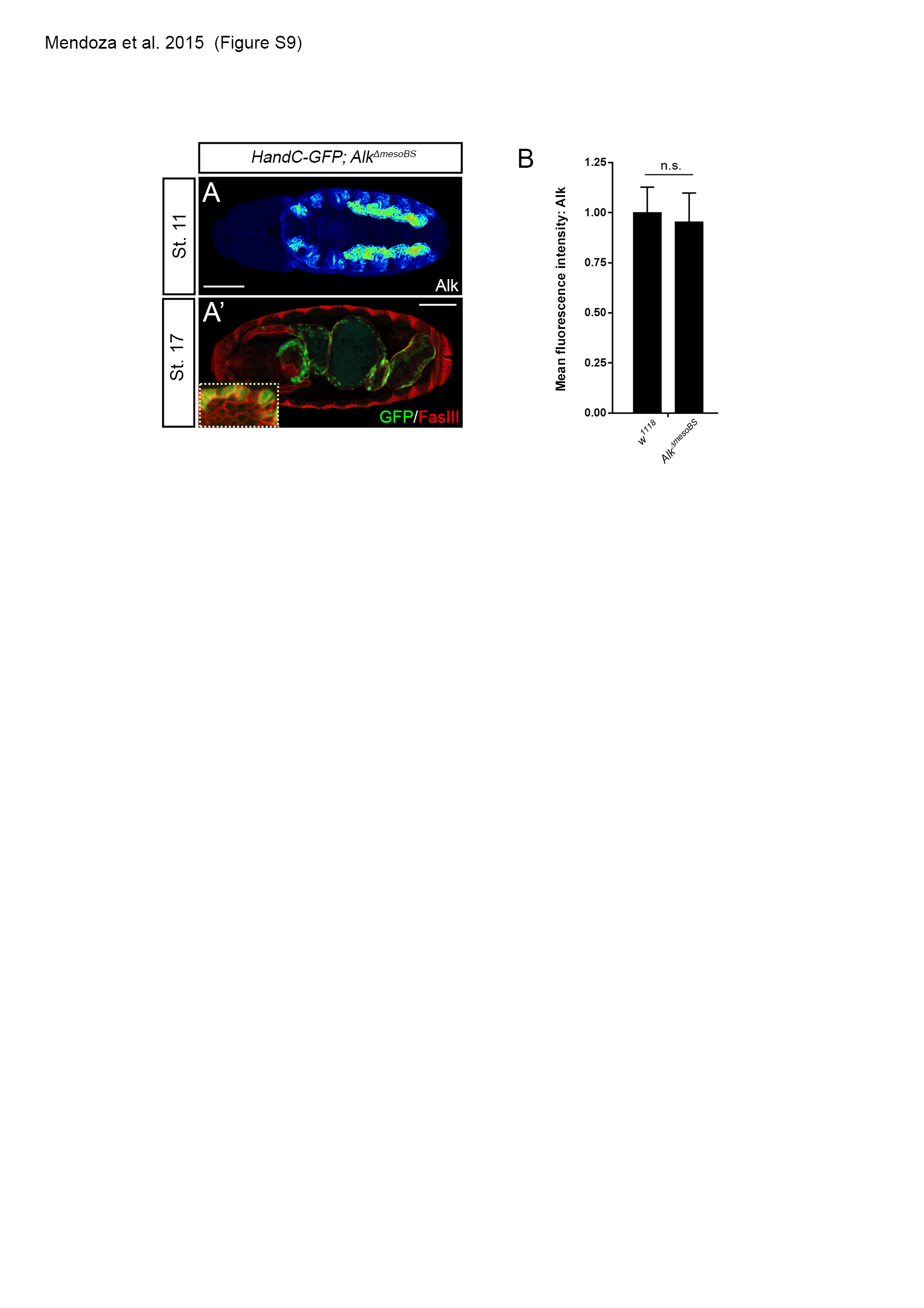

Supplement: S9 Fig — (A, A’) AlkΔmesoBS mutants do not show reduced levels of Alk protein in either the VM or epidermis (A, quantified in B). Furthermore, stage 16 homozygous mutant embryos show normal FC specification (inset, stage 11 embryo) and a chambered gut (A’, stage 16 embryo). (B) Quantification of Alk protein levels in AlkΔmesoBS mutants (n.s.–not significant; n = 10 animals per genotype). Scale bars: 50 μm. (TIF) [file pgen.1006617.s009.tif]

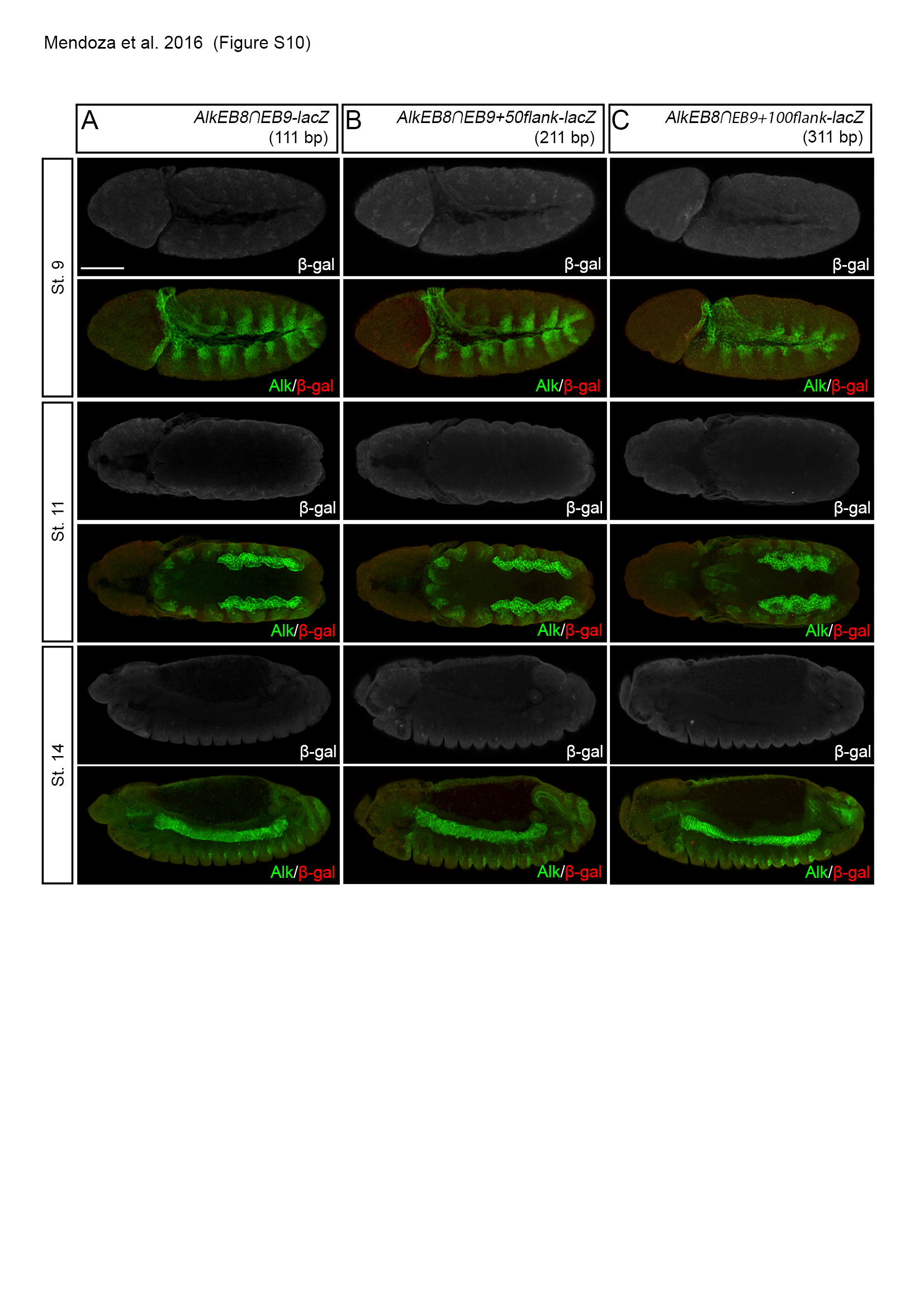

Supplement: S10 Fig — (A-C) lacZ reporters corresponding to the overlapping region between AlkEB8 and AlkEB9 were analyzed for reporter activity (schematically depicted in S1 Fig). None of the three transgenic sequences containing increasing portions of the overlapping region between AlkEB8 and AlkEB9, namely EB8∩9-lacZ (111 bp), EB8∩9+50flank -lacZ (211 bp) and EB8∩9+100flank-lacZ (311 bp) exhibit any detectable lacZ reporter activity during embryogenesis. Upper panels show lacZ reporter expression in white. Lower panels show a merged image of Alk protein shown in green and lacZ reporter expression in red. Scale bar: 50 μm. (TIF) [file pgen.1006617.s010.tif]

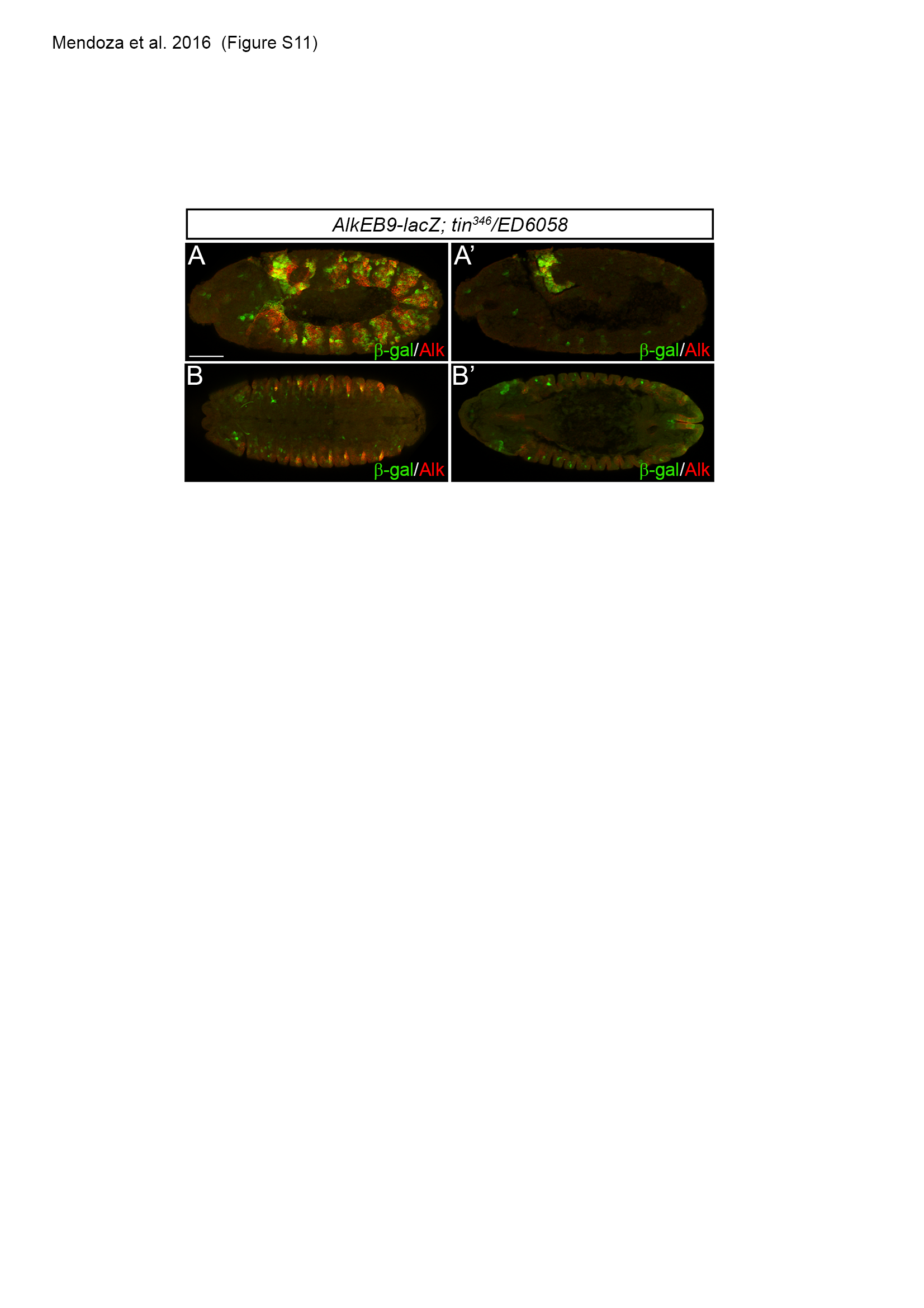

Supplement: S11 Fig — (A-A’) Expression of AlkEB9-lacZ in stage 11 tin/ED6058 mutant embryos. Lateral view of epidermal Alk (red) and β-gal (green) (A). Deeper view reveals no detectable Alk (red) or lacZ(green) expression (A’). (B-B’) Stage 14 tin/ED6058 mutant embryos do not exhibit VM structures (B’), however, both expression of AlkEB9-lacZ (green) and Alk protein (red) is visible in the epidermis (B). Scale bar: 50 μm. (TIF) [file pgen.1006617.s011.tif]

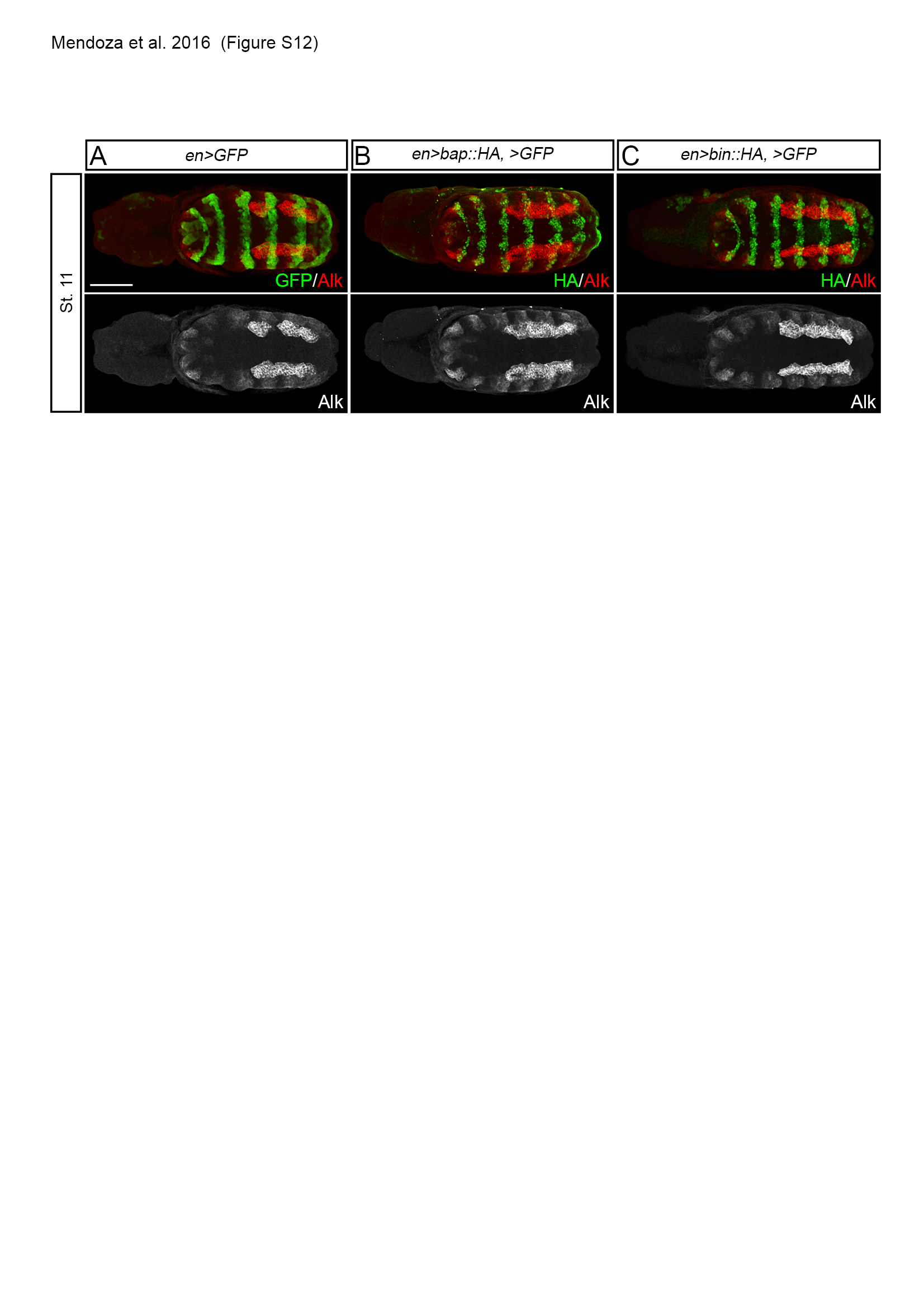

Supplement: S12 Fig — (A-C) Ectopic expression of either bap (B) or bin (C) in the epidermis with en-GAL4 does not result in a detectable increase in Alk protein levels. Alk protein shown in red, GFP in green (A), anti-HA in green (B, C). Scale bar: 50 μm. (TIF) [file pgen.1006617.s012.tif]

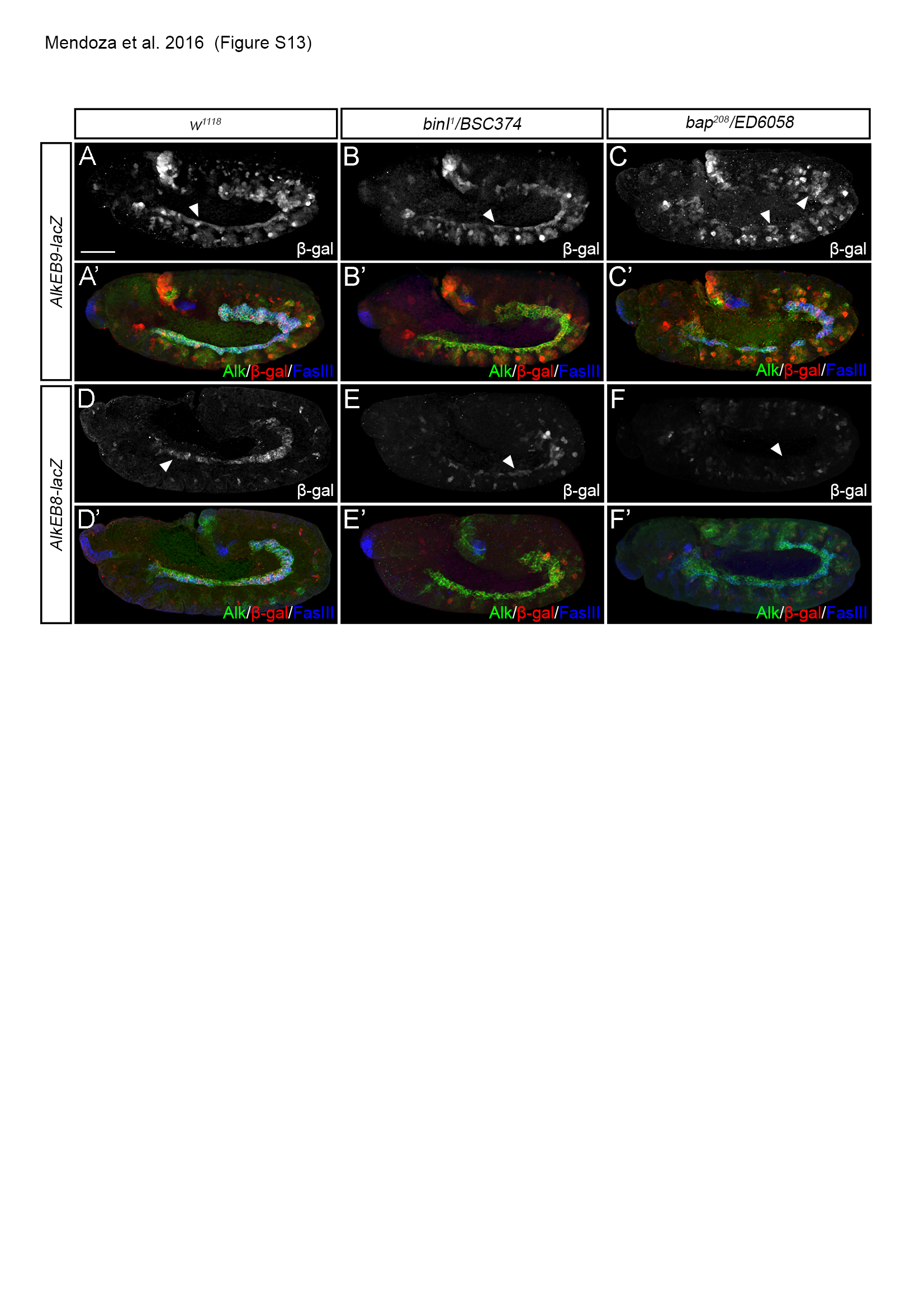

Supplement: S13 Fig — (A-C’) Expression of AlkEB9-lacZ in the VM is not altered in bin1/BSC374 or bap208/ED6058 embryos (arrowheads). (D-F’) In contrast, expression of AlkEB8-lacZ is mildly reduced in bin1/BSC374 animals and undetectable in bap208/ED6058 embryos (arrowheads). Alk protein is shown in green, lacZ reporter expression in red, FasIII shown in blue. Scale bar: 50 μm. (TIF) [file pgen.1006617.s013.tif]
